# Iron status influences non-alcoholic fatty liver disease in obesity through the gut microbiome

**DOI:** 10.1186/s40168-021-01052-7

**Published:** 2021-05-07

**Authors:** Jordi Mayneris-Perxachs, Marina Cardellini, Lesley Hoyles, Jèssica Latorre, Francesca Davato, José Maria Moreno-Navarrete, María Arnoriaga-Rodríguez, Matteo Serino, James Abbott, Richard H. Barton, Josep Puig, Xavier Fernández-Real, Wifredo Ricart, Christopher Tomlinson, Mark Woodbridge, Paolo Gentileschi, Sarah A. Butcher, Elaine Holmes, Jeremy K. Nicholson, Vicente Pérez-Brocal, Andrés Moya, Donald Mc Clain, Rémy Burcelin, Marc-Emmanuel Dumas, Massimo Federici, José-Manuel Fernández-Real

**Affiliations:** 1 Department of Endocrinology, Diabetes and Nutrition, Hospital of Girona “Dr Josep Trueta”, Girona, Spain; 2 Departament de Ciències Mèdiques, University of Girona, Girona and Biomedical Research Institute of Girona (IdibGi), Girona, Spain; 3 CIBERobn Pathophysiology of Obesity and Nutrition, Instituto de Salud Carlos III, Girona, Spain; 4 Department of Systems Medicine, University of Rome Tor Vergata, Via Montpellier 1, 00133 Rome, Italy; 5 Section of Biomolecular Medicine, Division of Systems Medicine, Department of Metabolism, Digestion and Reproduction, Imperial College London, Exhibition Road, London, SW7 2AZ UK; 6 Department of Bioscience, School of Science and Technology, Nottingham Trent University, Nottingham, NG11 8NS UK; 7 Institut National de la Santé et de la Recherche Médicale (INSERM), Toulouse, France; 8 Université Paul Sabatier (UPS), Unité Mixte de Recherche (UMR) 1048, Institut des Maladies Métaboliques et Cardiovasculaires (I2MC), Team 2: ‘Intestinal Risk Factors, Diabetes, Dyslipidemia, and Heart Failure’, 31432 Toulouse Cedex 4, France; 9 EPFL, Lausanne, Switzerland; 10 Department of Surgery, University of Rome Tor Vergata, Rome, Italy; 11 Unidad Mixta de Investigación en Genómica y Salud, Fundación para el Fomento de la Investigación Sanitaria y Biomédica de la Comunitat Valenciana (FISABIO) and Instituto de Biología Integrativa de Sistemas, Universitat de València and Consejo Superior de Investigaciones Científicas (CSIC), València, Spain; 12 CIBER en Epidemiología y Salud Pública (CIBERESP), Madrid, Spain; 13 Department of Internal Medicine, Wake Forest School of Medicine, Winston Salem, NC 27157 USA; 14 The W. G. Hefner Veterans Affairs Medical Center, Salisbury, NC 28144 USA; 15 Section of Genomic and Environmental Medicine, National Heart & Lung Institute, Imperial College London, Dovehouse Street, London, SW3 6LY UK; 16 European Genomic Institute for Diabetes, CNRS UMR 8199, INSERM UMR 1283, Institut Pasteur de Lille, Lille University Hospital, University of Lille, 59045 Lille, France; 17 McGill University and Genome Quebec Innovation Centre, 740 Doctor Penfield Avenue, Montréal, QC, H3A 0G1 Canada

**Keywords:** Systems medicine, Ferritin, Iron status, Gut microbiome, Non-alcoholic fatty liver disease, Shotgun sequencing, Metagenomics, Obesity

## Abstract

**Background:**

The gut microbiome and iron status are known to play a role in the pathophysiology of non-alcoholic fatty liver disease (NAFLD), although their complex interaction remains unclear.

**Results:**

Here, we applied an integrative systems medicine approach (faecal metagenomics, plasma and urine metabolomics, hepatic transcriptomics) in 2 well-characterised human cohorts of subjects with obesity (discovery *n* = 49 and validation *n* = 628) and an independent cohort formed by both individuals with and without obesity (*n* = 130), combined with in vitro and animal models. Serum ferritin levels, as a markers of liver iron stores, were positively associated with liver fat accumulation in parallel with lower gut microbial gene richness, composition and functionality. Specifically, ferritin had strong negative associations with the *Pasteurellaceae*, *Leuconostocaceae* and *Micrococcaea* families. It also had consistent negative associations with several *Veillonella*, *Bifidobacterium* and *Lactobacillus* species, but positive associations with *Bacteroides* and *Prevotella* spp. Notably, the ferritin-associated bacterial families had a strong correlation with iron-related liver genes. In addition, several bacterial functions related to iron metabolism (transport, chelation, heme and siderophore biosynthesis) and NAFLD (fatty acid and glutathione biosynthesis) were also associated with the host serum ferritin levels. This iron-related microbiome signature was linked to a transcriptomic and metabolomic signature associated to the degree of liver fat accumulation through hepatic glucose metabolism. In particular, we found a consistent association among serum ferritin, *Pasteurellaceae* and *Micrococcacea* families, bacterial functions involved in histidine transport, the host circulating histidine levels and the liver expression of *GYS2* and *SEC24B.* Serum ferritin was also related to bacterial glycine transporters, the host glycine serum levels and the liver expression of glycine transporters. The transcriptomic findings were replicated in human primary hepatocytes, where iron supplementation also led to triglycerides accumulation and induced the expression of lipid and iron metabolism genes in synergy with palmitic acid. We further explored the direct impact of the microbiome on iron metabolism and liver fact accumulation through transplantation of faecal microbiota into recipient’s mice. In line with the results in humans, transplantation from ‘high ferritin donors’ resulted in alterations in several genes related to iron metabolism and fatty acid accumulation in recipient’s mice.

**Conclusions:**

Altogether, a significant interplay among the gut microbiome, iron status and liver fat accumulation is revealed, with potential significance for target therapies.

Video abstract

**Supplementary Information:**

The online version contains supplementary material available at 10.1186/s40168-021-01052-7.

## Background

Non-alcoholic fatty liver disease (NAFLD) is a highly prevalent metabolic disease (the worldwide prevalence of NAFLD is 25.2% and increasing [1]) that can progress to cirrhosis and hepatocellular carcinoma, being a risk factor for the development of type 2 diabetes and cardiovascular disease. NAFLD is complex and multifactorial, with iron interacting with the development of NAFLD [2] through gluconeogenic signals [3]. In the liver, iron induces the synthesis and release of ferritin (an intracellular protein which stores iron), with its serum concentration proportional to body iron stores, and frequently increased in patients with NAFLD [4].

As the gut microbiome causally impacts the host phenome in hepatic liver fat accumulation [5], the composition of the gut microbiota could influence the impact of dietary iron on the development of NAFLD because this transition metal is a critical nutrient for both mammals and microorganisms [6]. Only ~ 5–15% of iron is absorbed and the remainder passes into the colon, where it is available to the gut microbiota [7]. The microbiota is also known to affect the absorption of key minerals, with iron being an important micronutrient in terms of its interactions with bacteria and the immune system [8].

Despite this emerging evidence suggesting a role of both the gut microbiome and iron in the pathogenesis of NAFLD, their complex cross-talk remains unclear. Therefore, in the present study, we applied an integrative systems medicine approach (faecal metagenomics, plasma and urine metabolomics, hepatic transcriptomics) in 3 well-characterised human cohorts, combined with in vitro and animal models, to characterize mechanisms responsible for the interaction between the gut microbiome and iron metabolism in NAFLD.

## Results

An overview of the study human cohorts and omics analyses pipeline can be found in Figure S1. Serum ferritin was measured in three cohorts: (a) a discovery cohort of subjects with obesity (*n* = 49); (b) a validation cohort of subjects with obesity from Italy and Spain (*n* = 628); and (c) an independent cohort of subjects with and without obesity from Spain (*n* = 130). Plasma and urine metabolomics were acquired in a subsample of both the discovery and the replication cohorts (plasma (n=48 and n=328) and urine (n=47 and n=322, respectively). The transcriptome was analysed in a subsample of the discovery and replication cohorts (*n* = 86). Finally, faecal samples from 56 women with obesity from the replication and validation cohort, and 130 subjects with and without obesity from the independent cohort were used to perform shotgun metagenomics sequencing.

### Increased serum ferritin is associated with liver fat accumulation and the gut microbiome composition and functionality

**Fig. 1:**
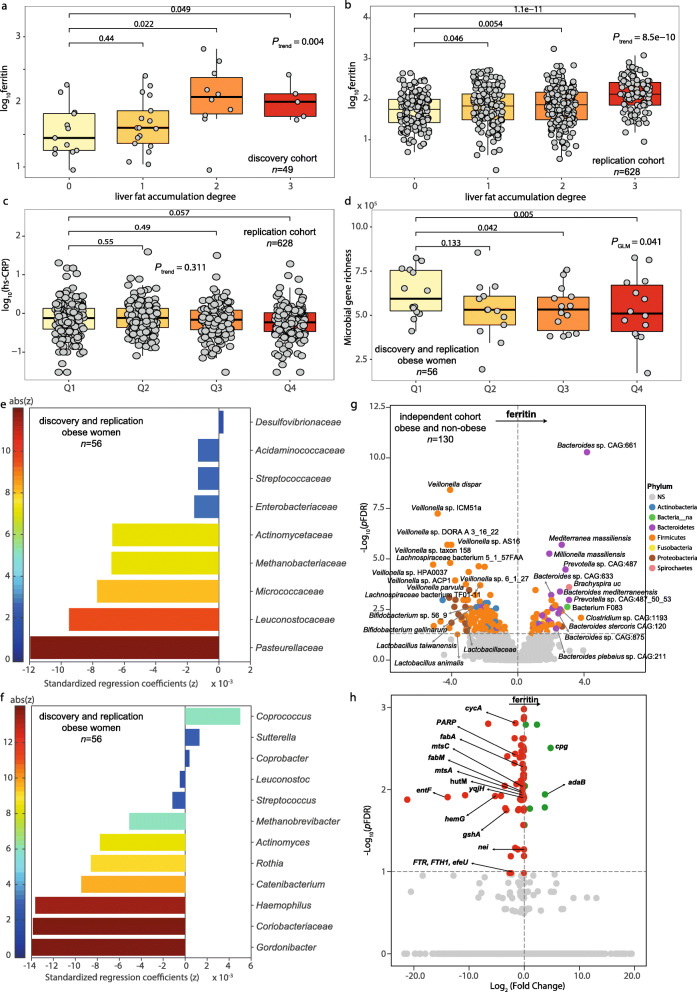
Association of serum ferritin with liver fat accumulation, gene richness and the gut microbiome composition. Association of serum ferritin with degree of liver fat accumulation in **a** the discovery and **b** replication cohorts (Mann-Kendall trend test and Wilcoxon tests). **c** Association of hs-CRP with serum ferritin quartiles in the replication cohort (Mann-Kendall trend test and Wilcoxon tests). **d** Association of microbial gene richness with ferritin quartiles in a subsample of obese women from the discovery and replication cohorts (generalized linear model GLM). **e** Bacterial families and **f** genera associated with serum ferritin in a subsample of obese women from the discovery and replication cohorts. Mnet penalized regression models were built on bacterial data including age, BMI, country and hs-CRP as covariates. **g** Volcano plot of differential bacterial abundance and **h** metagenome KEGG functions associated with ferritin as calculated from shotgun metagenomic sequencing in an independent cohort of obese and non-obese subjects, adjusting for age, BMI, sex and hs-CRP. Significantly different taxa are coloured according to phylum. *adaB*, methylated-DNA-[protein]-cysteine S-methyltransferase; *cpg*; glutamate carboxypeptidase; *cycA*; d-serine/d-alanine/glycine transporter; *fabA*, 3-hydroxyacyl-[acyl-carrier protein] dehydratase/trans-2-decenoyl-[acyl-carrier protein] isomerase; *fabM;* trans-2-decenoyl-[acyl-carrier protein] isomerase; *gshA*, glutamate-cysteine ligase; nei endonuclease VIII; *entF*, enterobactin synthetase component F; *FTR*, *FTH1*, *efeU*, high-affinity iron transporter; *hemG*; menaquinone-dependent protoporphyrinogen oxidase; *hutM,* histidine permease; *mtsC*; iron/zinc/manganese/copper transport system permease protein; *mtsA*; iron/zinc/manganese/copper transport system substrate-binding protein; *PARP*, poly [ADP-ribose] polymerase; *seqA*; negative modulator of initiation of replication; *yqjH*, ferric-chelate reductase (NADPH)

In both discovery and replication cohorts, serum ferritin increased with the severity of liver fat accumulation (Fig. 1a, b). No significant associations were found between high-sensitivity C-reactive protein (hs-CRP) and serum ferritin (Fig. 1c, Figure S2a,b) in any of the three cohorts. We also performed shotgun metagenomics, ^1^H NMR spectroscopy and transcriptomics to characterize the faecal microbiome, the biofluid metabolome and the liver transcriptome. Having processed > 5 Gb of metagenomic sequence data per individual, we derived taxonomy and gene richness as well as mapping and annotation of gene functions on the integrated gene catalog for a subsample (*n* = 56) of women with obesity from Italy and Spain [5]. Subjects within the highest ferritin quartiles (Q3 and Q4) had decreased gene richness compared with those in the lower ferritin quartile (Q1) (Fig. 1d). Consistently, multivariate penalized regression models adjusted for age, BMI, country and hs-CRP revealed a significant association between serum ferritin and the gut microbiome with significant decreases in families from the Firmicutes, Actinobacteria and Proteobacteria phyla, particularly *Pasteurellaceae*, *Leuconostocaceae* and *Micrococcaceae* (Fig. 1e,f). Similar results were obtained from multivariate orthogonal partial least squares (O-PLS) regression and posterior validation by univariate partial Spearman’s correlation (pSC) analyses adjusted by age, BMI, country and hs-CRP (Figure S3a–f). Notably, all identified ferritin-associated bacterial families (with the exception of *Leuconostocaceae*) had a strong correlation with iron-related genes such as *TFRC*, *HAMP*, *MitoNEET*, I*RP1* and ferroportin (*SLC40A1*), measured by quantitative qRT-PCR (Figure S4). We replicated these findings using DESeq2 analysis in an independent cohort of 130 subjects with and without obesity (Additional file 1: Table S1), in whom the majority of the associations between serum ferritin and bacterial families and genera were confirmed after adjustment for age, BMI, sex and hs-CRP (Fig. 1g, Figure S3g, h, Additional file 2: Table S2). The most consistent results were the negative associations of several *Veillonella*, *Bifidobacterium* and *Lactobacillus* species (from phyla Firmicutes and Actinobacteria) with serum ferritin levels, and positive associations with *Bacteroides and Prevotella* species (from phylum Bacteroidetes). When we analysed the data according to the obesity status, we found consistent negative associations of serum ferritin with *Lactobacillales*, *Pasteurellaceae*, *Streptococcaceae* and *Mycobacteriaceae* in both individuals with and without obesity. DESeq2 analysis at the species level also revealed consistent associations with *Veillonella* sp. *AS16*, *Veillonella* sp. *6*_*1*_*27*, *Lactobacillales*, *Streptococcus pneumoniae*, *Lachnospiraceae bacterium TF01*-*11*, *Bacteroides* sp. *GAC:633*, *Bacteroides coprophilus*, *Mediterranea massiliensis*, *Millionella massiliensis* and *Prevotella* sp. *CAG:487* in both subjects with and without obesity. Notably, the microbiome associated with hs-CRP was markedly different to that linked to serum ferritin (Figure S5a,b). In addition to the microbiome composition, their functionality was also evaluated by shotgun sequencing in this cohort. Remarkably, analysis of bacterial metagenomes based on KEGG functional annotation identified several bacterial functions related to iron and amino acids transport, glutathione metabolism, heme and siderophore biosynthesis, fatty acid biosynthesis and DNA replication and repair, associated with serum ferritin concentrations (Fig. 1h, Additional file 3: Table S3). Additional O-PLS regression analyses based on EggNOG functional annotations revealed similar results (Figure S5c, d and Additional file 4: Table S4).

### An iron-associated transcriptome signature is linked to the gut microbiome and liver fat accumulation

**Fig. 2:**
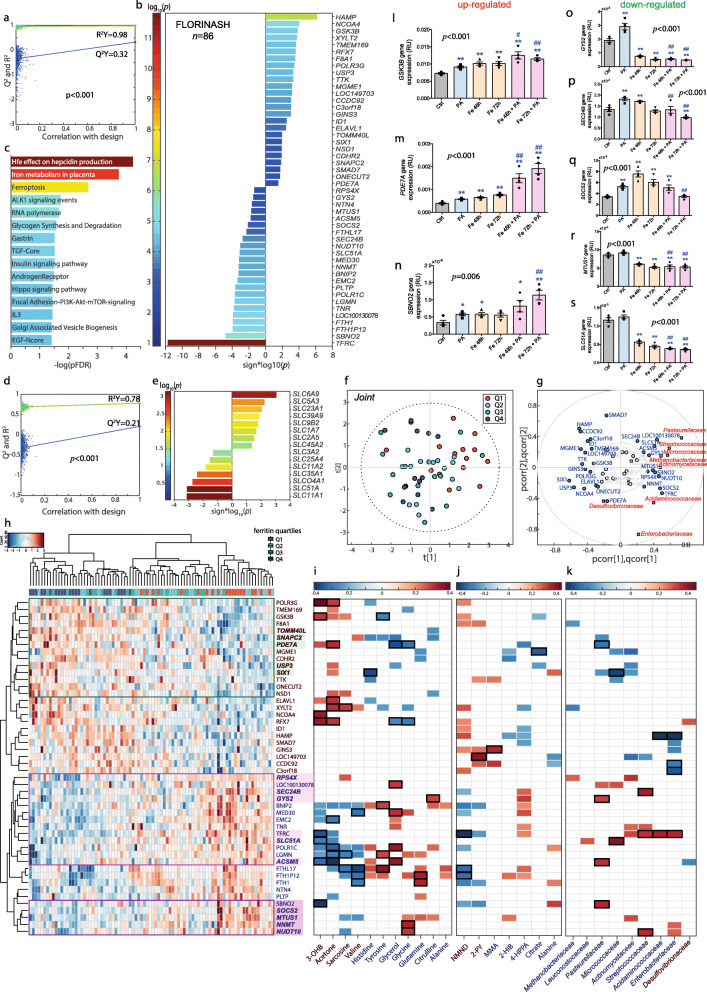
Association of transcriptomic data with serum ferritin. **a** Permutation test for the goodness-of-fit (*R*^2^*Y*) and goodness of prediction (*Q*^2^*Y*) obtained from the O-PLS model between serum ferritin and hepatic transcriptome in a subsample of the discovery and replication cohorts from Italy and Spain (*n* = 86). **b** Significant transcripts associated with serum ferritin after further validation of the O-PLS significant variables by pSC adjusting for age, sex, BMI and country. **c** Pathways significantly associated with serum ferritin based on mapping associated transcripts by over-representation analysis with hypergeometric test. **d** Permutation tests for the O-PLS model between serum ferritin and SLCs (*n* = 86). **e** Significant SLCs associated with serum ferritin after further validation of the O-PLS results by pSC adjusting for age, sex, BMI, and country. **f** O2-PLS scores for the joint variation between microbial families and genes associated with serum ferritin. A model with 2 predictive components, and 1 orthogonal component for the genes and bacterial families blocks, was constructed based on 7-fold cross-validation. **g** O2-PLS joint loadings plots, where pcorr represents the correlation-scaled loadings from the gene block and qcorr represents the correlation-scaled loadings from the bacterial families block. **h** Heatmap displaying *z*-scores of the ferritin-associated transcripts for each subject. Clustering was based on Euclidean distances and Ward linkage. Genes associated with liver fat accumulation from O-PLS modelling are highlighted in bold, whereas those associated with bacterial families from O2-PLS modelling are highlighted in colour boxes. **i** Heatmap for the pSC adjusted by age, BMI, sex, and country between ferritin-associated plasma and **j** urine metabolites with ferritin-associated transcripts (*n* = 86). **k** Significant (*p* < 0.05) pSC adjusted for age, BMI and country, between ferritin-associated families and transcripts (*n* = 56). Only significant associations (*p* < 0.05) are displayed. Significant associations after a pFDR correction (*p*FDR < 0.05) are highlighted with a black box. **l–n** Expression of upregulated (*GSK3B*, *PDE7A*, *SBNO2*) and **o**–**s** downregulated genes (*GYS2*, *SEC24B*, *SOCS2*, *MTUS1* and *SLC51A*) in human primary hepatocytes after treatment with iron and palmitic acid. Data are mean ± SEM. Comparisons by one-way ANOVA. *p < 0.05, **p < 0.01, ***p < 0.001 compared to control group based on *t* test. ^#^*p* < 0.05, ^##^*p* < 0.01, ^###^*p* < 0.001 compared to PA group based on *t* test. Ctrl, control group; PA, palmitic acid; Fe48h, pre-treatment iron 50 μM for 48h; Fe72h, pre-treatment iron 50 μM for 72h; Fe48h + PA, pre-treatment iron 50 μM for 48h + palmitic acid 200 μM for 24 h; Fe72h + PA, pre-treatment iron 50 μM for 72 h + palmitic acid 200 μM for 24 h

We then explored the associations of serum ferritin with the liver transcriptome in a subsample (*n* = 86) of the discovery and replication cohorts from Italy and Spain. Out of the 48 mRNAs identified from an O-PLS model (Fig. 2a), transferrin receptor (*TFRC*, *p*FDR < 1.0 × 10^−10^), hepcidin antimicrobial peptide (*HAMP*, *p*FDR = 1.95 × 10^−5^), *NCOA4* (*p*FDR = 0.05) and ferritin heavy chain (*FTH1*, *p*FDR = 0.003), all involved in iron status [9], were the mRNAs most associated with serum ferritin after further individual validation by pSC (Fig. 2b). Enrichment analyses highlighted a significant over-representation of pathways associated with iron and glucose metabolism (Fig. 2c). We further investigated the association between the expression of several solute carrier transporters (SLCs) and the serum ferritin concentration (Fig. 2d, e). *SLC51A*, *SLC11A1* and *SLC6A9* had the strongest associations with serum ferritin. Afterwards, integrating metagenomic and transcriptomic results, we identified iron-associated transcriptome signatures linked to the microbiome and the degree of liver fat accumulation (Fig. 2f–k, Figure S6). From O2-PLS multivariate integration between ferritin-associated bacterial families and transcripts, hierarchical clustering analysis and univariate pSC, we identified a clear cluster comprising *NUDT10*, *NNMT*, *MTUS1*, *SOCS2* and *SBNO2*, downregulated with increased ferritin levels (Fig. 2h) and correlated to variation in different bacterial families that were themselves linked to serum ferritin (Fig. 2g, k). In a second cluster, the expression of *SEC24B*, *GYS2*, *SLC51A*, *TFRC, RPSX5*, *LOC100130078* and *ACSM5* was also mirrored by these families. A third cluster of genes positively associated with ferritin (*USP3*, *SIX1*, *PDE7A* and *SNAPC2*) also anti-correlated with those bacterial families. Remarkably, the expression of most of these bacterial-associated genes, changed in proportion to both serum ferritin levels and liver fat accumulation (Figure S6).

### The transcriptome signature was replicated in human primary hepatocytes

We then sought to validate the transcriptome findings by studying the effects of iron and palmitic acid (PA), a trigger of hepatic fat accumulation [10], in human primary hepatocytes. We found that iron, PA and PA supplementation in cells pretreated with iron led to triglyceride accumulation in primary human hepatocytes (Figure S7a, b). Notably, iron induced a striking increase in the expression of lipid metabolism genes (*FABP4*, *FABP5*, *FATP5*) and of the fatty acid transporter CD36 in synergy with PA (Figure S7c–f) in parallel to upregulated iron-related genes (FTL and FTH) (Figure S7g, h).

Strikingly, PA supplementation in cells pretreated with iron downregulated the expression of most of the identified genes associated negatively with serum ferritin (*GY2*, *SEC24B*, *MTUS1*, *SOCS2*, *SLC51A*) compared to PA or iron alone (Fig. 2l–s) in parallel to fat accumulation, confirming the associations observed in subjects with different degrees of hepatic fat accumulation. The exception was *SBNO2*, known to be increased in proinflammatory responses. Conversely, genes associated positively with serum ferritin (*PDE7A*) and also with liver steatosis increased significantly after iron exposure.

### Metabolomics identifies gluconeogenic substrates and ketone bodies connected with the iron-related microbiome and transcriptomic signatures

**Fig. 3:**
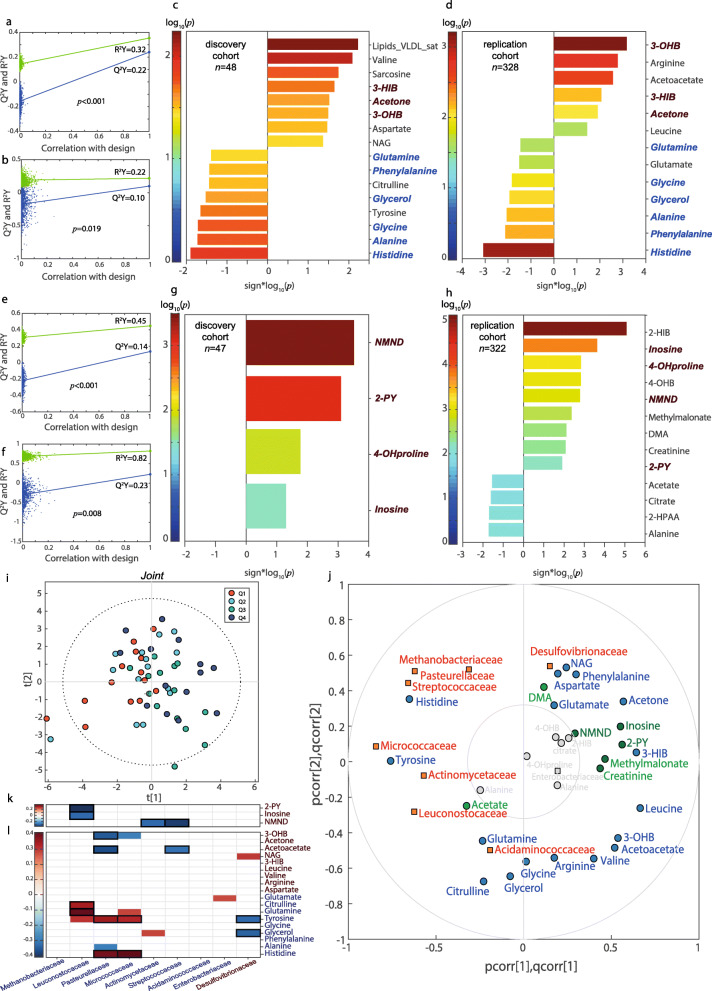
Associations of metabolomic data with serum ferritin. Permutation tests for the goodness-of-fit (*R*^2^*Y*) and goodness of prediction (*Q*^2^*Y*) obtained from the O-PLS model between serum ferritin and **a** the serum (*n* = 48) and **e** urine metabolome (*n* = 47) in the discovery cohort, and **b** the serum (*n* = 328) and **f** urine metabolome (*n* = 322) in the replication cohort. Significant **c**, **d** serum and **g**, **h** urine metabolites associated with serum ferritin after further validation of O-PLS identified metabolites by pSC adjusting for age, sex, BMI and country. **i** O2-PLS scores for the joint variation between plasma and urine metabolites and microbial families associated with serum ferritin. A model with 2 predictive components, and 0 and 1 orthogonal component for the metabolites and bacterial families blocks, was constructed based on 7-fold cross-validation. **j** O2-PLS joint loadings plots, where pcorr represents the correlation-scaled loadings from the gene block and qcorr represents the correlation-scaled loadings from the bacterial families block. **k** Heatmap for the pSC adjusted by age, BMI and country between ferritin-associated urine and **l** plasma metabolites with ferritin-associated bacterial families (*n* = 56). Only significant associations (*p* < 0.05) are displayed. Significant associations after a pFDR correction (*p*FDR < 0.05) are highlighted with a black box

We then performed discovery and replication metabolome-wide association studies (MWAS) for ferritin in serum (Fig. 3a–d) and urine (Fig. 3e–h) using O-PLS multivariate regressions confirmed by pSC. We identified several metabolites, such as ketone bodies and gluconeogenic substrates, in both discovery (*n* = 48 for plasma; *n* = 47 for urine) and replication (*n* = 328 for plasma; *n* = 322 for urine) cohorts associated to the identified hepatic transcriptome signatures (Fig. 2i, j) linked to the microbiome and severity of NAFLD. Integration of ferritin-associated metabolites and bacterial families by O2-PLS regression (Fig. 3i, j) revealed strong positive associations between histidine, tyrosine, citrulline and glutamine and bacterial families negatively associated with serum ferritin (Fig. 3j–l). These families also had strong negative associations with ketone bodies (3-hydroxybutyrate (3-OHB) and acetoacetate).

### Iron influences the gut microbiome composition

**Fig. 4:**
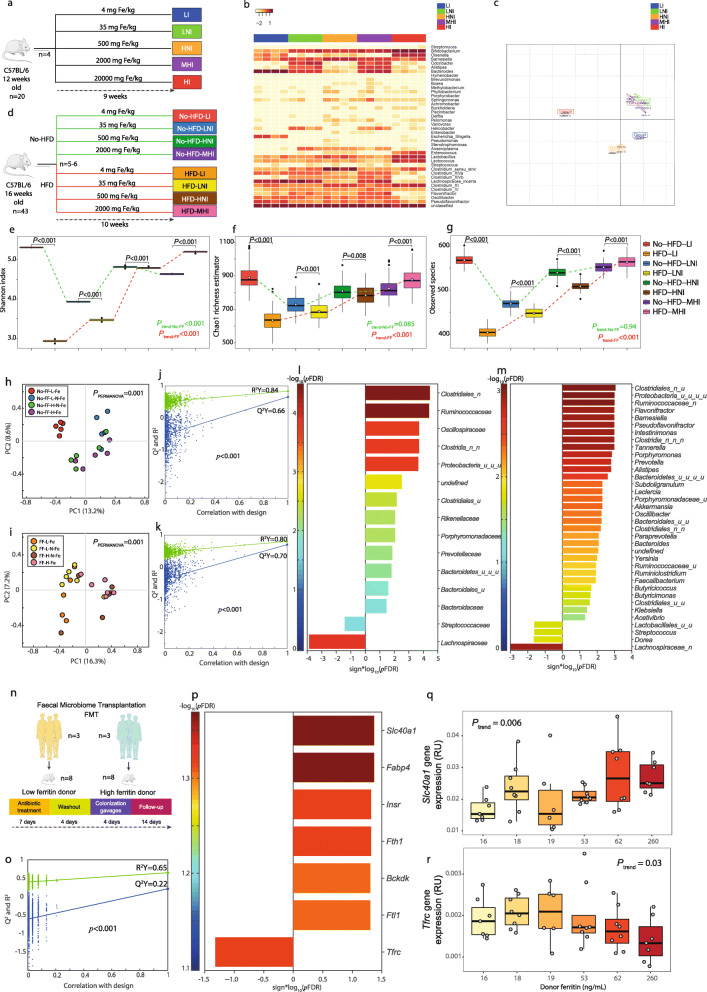
Validation studies in primary hepatocytes and FMT mice. **a** Scheme of the experimental design for study 1. Mice were fed for 9 weeks diets containing low- (LI), low-normal- (LNI), high-normal- (HNI), moderately high- (MHI) and high- (HI) iron doses. **b** Heatmap displaying genus relative abundances for each mouse. **c** Principal coordinate analysis (PCoA) depicting dissimilarities between groups based on unifrac distance metrics. **d** Scheme of the experimental design for study 2. Mice were fed either a high fat diet (HFD) or a no-HFD diet containing four different iron doses (LI, LNI, HNI, MHI) for 10 weeks. **e** Variations in the Shannon diversity index, **f** Chao1 richness estimator and **g** observed species of mice fed either a HFD or a no-HFD with different iron doses (LI, LNI, HNI, MHI). **h** PCoA based on Canberra distance metric for the no-HFD-fed mice and **i** the HFD-fed mice with different iron doses. Differences in microbial composition between iron doses for each diet were assessed by PERMANOVA using 999 permutations. **j**, **k** Permutation tests for the O-PLS models between iron dose and bacterial families or genera in HFD-fed mice, respectively. **l** Significant families and **m** genera identified from O-PLS regression loadings to be associated with iron dose. **n** Scheme of the experimental design for study 3. Low-ferritin (*n* = 3) and high-ferritin (*n* = 3) microbiota human donors were selected and for each donor their faecal samples were transplanted *n* = 6–8 mice after antibiotic treatment. After 14 days following colonization gavage mice were sacrificed and iron and liver fat accumulation-related genes (*n* = 22) were measured by PCR. **o** Permutation test for the O-PLS-DA model between mice genes and the human donor group (low- or high- ferritin). **p** Significant mouse genes associated with donor group from O-PLS-DA regression loadings. **q** Ferroportin (*Slc40a1*) and **r** *Tfrc* expression according to the donor ferritin concentration

To validate the cross-talk between iron status and the microbiome uncovered in humans, we first tested whether the dietary iron content impacts the microbiome in vivo in the mouse (Fig. 4a). Using 16S rRNA gene amplicon sequencing, we showed that variation in dietary iron dramatically reshapes the composition of the gut microbiota (Fig. 4b, c). Then, we characterised the impact of a high-fat diet vs. control diet with different iron contents in mice using metagenomics (Fig. 4d). Bacterial biodiversity and observed species changed dramatically according to fat and iron content of the diet (Fig. 4e–g). While a high-fat diet decreased bacterial biodiversity under a low iron diet, the opposite as found in diets with high iron content. Principal coordinate analyses revealed different microbial community compositions depending on the iron content in each diet (Fig. 4h, i). Interestingly, the differences in the microbial composition between the high-fat and control diets decreased with iron content, becoming non-significant at high iron levels (Figure S8a–d). Several families and genera in the phylum Firmicutes inversely associated with serum ferritin in patients were confirmed to be influenced accordingly by the iron content of the mice diet in O-PLS models (Fig. 4j–m).

### The gut microbiome affects iron metabolism

After showing that iron availability greatly influences the gut bacterial ecosystem, we evaluated whether the microbiota on its own might affect iron status. We leveraged our previous mouse study showing that faecal microbiota transplantation (FMT) triggered hepatic fat accumulation [5] to evaluate the relationship between human donor ferritin levels and iron-related genes in recipient mice (Fig. 4n). O-PLS-discriminant analysis revealed that microbiota from the ‘high ferritin donors’ group resulted in alterations in genes related to iron metabolism, with increases in *ftl1*, *fth1* and s*lc40a1*, in parallel to decreased *tfrc* (Fig. 4o–r), which is in line with the transcriptomic results in humans and the in vitro results from human primary hepatocytes. Also, in agreement with the in vitro results, the microbiota from ‘high ferritin donors’ also increased the expression of *fabp4* in recipient’s mice livers, showing the effect ferritin-associated microbiota on liver lipid accumulation.

## Discussion

In the current study, we evaluated the contribution of the gut microbiota in iron status and liver fat accumulation in a discovery and validation cohort of patients with obesity and an additional independent cohort of individuals with and without. We further identified a potential role of the microbiome as a regulator of iron status controlling hepatic fat deposition in animal models. Serum ferritin levels were positively associated with liver fat accumulation in parallel to a decrease in several bacterial families. At the same time, these ferritin-related families were associated with liver genes involved in iron metabolism. The *Pasteurellaceae* family had the strongest negative association with the serum ferritin levels, which is consistent with their reliance on utilizing iron from host transferrin for growth and survival [11]. Recently, hepatic lipid levels, including bile acids, have been recently negatively correlated with *Micrococcaceae* in diabetic mice [12], and the *Desulfovibrionaceae* family has been associated to overfeeding-induced fatty liver [13]. Consistent with our results, increases in the caecal contents of *Coprococcus* were identified in rats supplemented with iron, which were suggested to mediate oxidative stress and histopathological alterations observed in the liver of these animals [14]. Also in agreement with our results, low serum ferritin concentrations coexisted with decreased abundance of *Veillonella* species in ulcerative colitis patients receiving FMT from healthy donors [15], while *Veillonella* genus abundance was dose-dependently enriched after improvement of steatosis in NASH patients [16]*.* In addition, those with > 70% reduction in liver fat had a trend towards reduction of *Methanobrevibacter*, which we also found negatively associated with serum ferritin levels. Significant and drastic increases in *Bacteroides* and *Prevotella* have been observed in obese and NASH individuals, while a progressive decrease in the abundance of *Bifidobacterium* was observed from healthy to NASH groups [17]. Finally, lack of iron requirements in lactic acid bacteria is in agreement with the negative associations observed among several *Lactobacillus* species and the serum ferritin levels.

Although we used ferritin as a marker of iron store, we must take into account that it is also an acute-phase reactant which is increased under inflammatory conditions [18]. Given that our study subjects were all obese, and low-grade chronic inflammation is a hallmark of obesity [19], inflammation could have an influence on ferritin levels. Importantly, we did not find any significant association between inflammatory markers (hs-CRP) and serum ferritin, suggesting that serum ferritin was measuring iron stores in our cohorts. To further rule out the effect of inflammation in the identified associations, we adjusted our analysis by hs-CRP. Notably, the microbiome associated with hs-CRP was markedly different to that linked to serum ferritin, confirming that the observed microbiome-ferritin associations were independent of inflammation.

In addition to identifying an iron-associated microbiome signature, we also evaluated the microbiome functionality. Different bacterial pathways were associated with iron stores in the host, including heme and siderophore (iron binding molecules) biosynthesis, iron transport, glutathione metabolism and DNA replication and repair (known to be iron-dependent). Of note, the bacterial cytochrome b561 function was strongly negatively associated with serum ferritin levels. As cytochrome b561 is known to be involved in iron absorption [20], this finding suggests that the bacterial cytochrome b561 competes with the enzyme present in the intestine influencing iron uptake. In line, the expression of several bacterial iron transport and chelation functions (*FTR*, *FTH1*, *efeU*; *yqjH*; *mtsC*; and *mtsA*) was reduced in subjects with high serum ferritin levels. Similarly, bacterial enzymes involved in heme biosynthesis (uroporphyrinogen decarboxylase; and *hemG*, menaquinone-dependent protoporphyrinogen oxidase) and siderophore biosynthesis (*entF*, enterobactin synthetase component F) were also strongly negatively associated with serum ferritin. It is also worth noting the strong associations of host serum ferritin levels with bacterial functions involved in glutathione biosynthesis (*gshA*, glutamate-cysteine ligase) and glutathione precursors (*cpg*, glutamate carboxypeptidase). Interestingly, the plasma and liver levels of glutathione are depleted in NAFLD patients and altered glutathione metabolism has been identified as a prevailing feature in NAFLD [21].

We also evaluated the associations of serum ferritin with the liver transcriptome and the serum and urine metabolome. Remarkably, we identified iron-associated transcriptome signatures linked to the microbiome and the degree of liver fat accumulation, which we confirmed by supplementing human primary hepatocytes with iron and PA, a trigger of hepatic fat accumulation [10]. Importantly, excessive gluconeogenesis has been previously associated with NAFLD in humans [22] and iron has been also shown to influence gluconeogenic signals [3]. In line with these previous results, we found alterations of genes involved in glucose metabolism. Thus, from those mRNAs involved in the microbiome-associated transcriptomic signature of iron (also linked to liver fat accumulation), *GYS2* (which catalyses the rate-limiting step in the synthesis of glycogen) showed the strongest negative association with NAFLD (Figure S6). Disruption of *GYS2* is known to result in impaired glucose deposition and hepatic insulin resistance and liver fat accumulation in mice by changing de novo lipogenesis through increased expression of SREBP1c [23]. Insulin also signals to SREBP1 through inhibition of GSK3, which we found positively correlated with ferritin levels. In line with these results, we found an upregulation of *GSK3B* after treating human primary hepatocytes with iron or palmitic acid, which was exacerbated after co-treatment. Interestingly, we found a negative correlation between ferritin and insulin action measured through euglycemic hyperinsulinemic clamp (*r* = − 0.31, *p* = 8.9e-4). *GYS2* clustered with *SEC24B*, which is responsible for the ER-to-Golgi transport of proteins, and disrupted ER-to-Golgi trafficking has shown to contribute to ER stress, hepatic injury and NAFLD [24, 25]. These transcriptomics findings were supported by metabolomics results. Therefore, we identified some ferritin-associated metabolites (sarcosine, citrulline, glutamate) that have been previously linked to iron-induced impairment of glucose metabolism [26]. In agreement, we found that subjects with higher ferritin concentrations had lower serum levels of glutamine, alanine and glycerol, the main substrates used for liver gluconeogenesis, which is documented by liver transcriptomics (Fig. 2b and Figure S6). Notably, glutamine had a strong positive association with *Leuconostocaceae*, one of the bacterial families most negatively associated with serum ferritin levels. However, the most consistent effect was the negative association of serum ferritin with histidine levels in both the discovery and replication cohorts. Histidine has shown to supress hepatic gluconeogenesis by activation of STAT3 independent of central insulin action [27]. Noticeably, the host serum ferritin levels were strongly associated with the bacterial function histidine permease (*hutM*), and the *Pasteurellaceae* and *Micrococcaceae* families, both also positively associated with the liver expression of *GYS2*. These families were also strongly associated with the *SLC51A* expression*.* This gene is involved in bile acid transport and has recently been associated with NASH [28]. Importantly, the gut microbiota can regulate the pool size and composition of bile acids [29], which play an important role in NAFLD pathogenesis and progression [30]. Interestingly, most bile acids are conjugated to glycine and we identified glycine as negatively associated with serum ferritin (Fig. 3c, d). In addition, glycine is a key rate-limiting component of heme biosynthesis, mainly supplied by the glycine transporter 1 (GLYT1) encoded by *SLC6A9*, which was positively associated with serum ferritin (Fig. 2d, e). This increased glycine demand may account for the lower serum levels associated with high ferritin concentrations. In addition, glycine is the limiting substrate in glutathione synthesis from glutamate in subjects with NAFLD [21]. Remarkably, we found a strong negative association between serum ferritin levels and the expression of the bacterial glycine transporter (*cycA*) and glutamate-cysteine ligase (*gshA*), the first enzyme in the glutathione biosynthetic pathway. Finally, serum acetone and 3-OHB were also positively and consistently associated with serum ferritin concentration, in line with hyperinsulinemia resulting in shifted energy supply from glucose to ketone bodies in NAFLD in parallel with increased circulating levels of the latter [31]. The serum 3-OHB levels also had a strong negative correlation with the liver expression of *SLC51A* and *SBNO2*, both positively associated with *Pasteurellaceae* and *Micrococcaceae* families. Consistently, *SBNO2* expression increased markedly following LPS-induced systemic endotoxemia [32]. Conversely, the control of LPS signalling by *SOCS2*, another negative inflammation regular, is minimal [33], which is also consistent with the lack of associations that we observed among bacterial families and the liver expression of this gene.

We sought to validate these results by treating human primary hepatocytes with iron and palmitic acid. For those gene transcripts that were positively associated with serum ferritin in the discovery cohort (*GSK3B*, *PDE7A*) including subjects with different degrees of liver fat accumulation, we found a consistent upregulation of these genes after treatment with either palmitic acid or iron. Co-treatment with palmitic acid and iron exacerbated these effects. Some genes negatively associated with serum ferritin and steatosis degree, were consistently downregulated after treatment with iron or iron + palmitic acid (*SLC51A*, *MTUS1*). However, the results obtained for other genes negatively associated with serum ferritin in this cohort with steatosis seemed counterintuitive (*GYS2*, *SEC24B*, *SOCS2*). Hence, contrary to what we expected, treatment with iron and/or palmitic acid led to an upregulation of these genes in human primary hepatocytes. However, co-treatment with both iron and palmitic downregulated the expression of these genes, which is in line with the results observed in the discovery cohort. We hypothesize that this downregulation could arise from an ‘hormesis effect’, i.e., an adaptative compensatory process following an initial disruption of homeostasis, to compensate the initial disruptions in gene expression induced by palmitic acid or iron alone.

Our results, based on the identification several bacterial species and metagenome functions involved in iron metabolism and NAFLD, suggested a direct impact of the microbiome on iron metabolism. Hence, we further explored the potential causative role of the gut microbiome on iron metabolism and liver fact accumulation using a mouse FMT experiment. Faecal microbiome transplantation from ‘high ferritin donors’ into recipient mice increased the expression of several genes involved in iron metabolism as well as that of genes that promote fatty acid accumulation such as *fabp4*, which is consistent with results observed after treating human primary hepatocytes with iron. Conversely, microbiota from ‘low ferritin donors’ increased the expression of iron-related *Tfrc.* This is agreement with our findings in human subjects, where we found strong positive associations among *Actinomycetaceae*, *Acidaminococcacea* and *Enterobacteriaceae* families (both increased in the microbiota of subjects with low serum ferritin levels) and the host liver *TFRC* expression*.* These results are also consistent with the negative association found among metagenome functions related to fatty acid biosynthesis (*fabM* and *fabA*) and the host serum ferritin concentrations. This could reflect a possible use of fatty acids from the host by the microbiota, avoiding the process of de novo synthesis. Therefore, we showed that the microbiota itself could recapitulate in recipient mice the phenomic hallmarks of iron metabolism from the human donor, thereby impacting liver fat accumulation in the long term.

The current study presents some limitations. A minority of the bacterial population might have a role in NAFLD development but could remain undetected by metagenomics. On the other hand, the FMT experiment has also its limitations because some strictly anaerobic bacterial species might have a role in NAFLD development but are lost during sample collection, storage and manipulation. We showed changes in the expression of iron-related genes, but the impact of these changes on iron levels of mice was not evaluated. Therefore, the causal role of the microbiome as a regulator of iron status needs to be further confirmed with the measurement of circulating iron makers in these mice. Finally, we showed iron supplementation impacts on the microbiome composition and that bacterial biodiversity and observed species changed dramatically according to fat and iron content of the diet (Fig. 4e–g). It needs to be further investigated whether iron supplementation impacts on fat deposition in the liver in mice under different feeding regimes.

## Conclusions

In conclusion, combining a comprehensive systems medicine approach with validations in independent cohorts and causality assessment in pre-clinical models, our findings demonstrate a significant cross-talk among gut microbiota, iron status and liver fat accumulation. In particular, we uncover microbiome- and iron-linked metabolomic and transcriptomic signatures involving imbalances in gluconeogenic metabolites, ketone bodies and cellular transport, which altogether modulate liver fat accumulation. This work highlights the crucial importance of the interplay between micronutrients, microbiome and host homeostasis in general [34] and the Microbiome-Iron-Liver fat axis in particular, thereby disclosing potential targets for therapy.

## Patients and methods

Detailed methods for the animals studies, 1H-NMR metabolomics, liver transcriptomics and faecal 16S rRNA and metagenomics sequencing can be found in Additional file 5: Supplementary methods.

### Patient recruitment and sample processing

The discovery cohort included *n* = 49 obese patients aged 24 to 63 years old at the Endocrinology Service of the Hospital Universitari de Girona Dr Josep Trueta (Girona, Spain). The replication cohort comprised *n* = 628 obese patients aged 20 to 67 years old at the Endocrinology Service of the Hospital Universitari de Girona Dr Josep Trueta (*n* = 287) and at the Center for Atherosclerosis of Policlinico Tor Vergata University of Rome (Rome, Italy; *n* = 341). Sample size was not determined by statistical methods and is comparable to other studies in the field [35–37]. All subjects gave written informed consent, validated and approved by the ethical committee of the Hospital Universitari Dr Josep Trueta (Comitè d’Ètica d’Investigació Clínica, approval number 2009 046) and Policlinico Tor Vergata University of Rome (Comitato Etico Indipendente, approval number 28-05-2009). Inclusion criteria included Caucasian origin, stable body weight 3 months before the study, free of any infection 1 month preceding the study and absence of any systemic disease. Exclusion criteria were the following: presence of liver disease (specifically tumoural disease and hepatitis C virus infection) and thyroid dysfunction (based on biochemical work-up), alcohol consumption (> 20 g/day), hepatitis B (anti-HD virus antibodies), drug-induced liver injury (using a drug questionnaire).

A third independent cohort included obese (BMI ≥ 30 kg/m^2^) patients and age- and sex-matches non-obese subjects (BMI 18.5–< 30 kg/m^2^) aged 27–67 years old, recruited at the Endocrinology Service of the Hospital Universitari de Girona Dr Josep Trueta (Girona, Spain). Exclusion included type 2 diabetes mellitus, chronic inflammatory systemic diseases, acute or chronic infections in the previous month; use of antibiotic, antifungal, antiviral or treatment with proton-pump inhibitors; severe disorders of eating behaviour or major psychiatric antecedents; neurological diseases, history of trauma or injured brain, language disorders; and excessive alcohol intake (≥ 40 g OH/day in women or 80 g OH/day in men). All subjects gave written informed consent, validated and approved by the ethical committee of the Hospital Universitari Dr Josep Trueta.

Stool, plasma and urine samples from all subjects were obtained during the week before elective gastric bypass surgery, during which the liver biopsy was sampled. All samples were stored at − 80 °C. Liver samples were collected in RNAlater, fragmented and immediately flash frozen in liquid nitrogen before storage at − 80 °C.

### Hepatic steatosis

An ultrasound system with a 3.5 MHz convex transducer (Siemens Acuson S2000, Mochida Siemens Medical System, Tokyo, Japan) was used to scan the liver. Hepatic steatosis was defined as absent (grade 0: < 5% steatosis), mild (grade 1: 5–33% steatosis), moderate (grade 2: > 33–66% steatosis) or severe (grade 3: > 66% steatosis) using the scoring system for NAFLD [38]. Images were independently evaluated by two radiologists blinded to clinical and laboratory data [39].

### Liver histology

Liver biopsies were previously obtained for *n* = 86 patients who underwent bariatric surgery [5]. The investigators were blind to group allocations. Liver biopsies were analysed by a single pathologist expert in hepatic pathology. For each liver sample, haematoxylin and eosin, reticulin and Masson’s trichrome staining were performed. Hepatic steatosis grade was determined according to the scoring system for NAFLD [38].

### Serum ferritin

Serum ferritin in both discovery and replication cohorts was measured by microparticle enzyme immunoassay (AxSYMTM; Abbot Laboratories) with intra- and inter-assay CVs < 6%.

### ^1^H nuclear magnetic resonance spectroscopy-base metabolic profiling

Spectroscopic analysis of urine (*n* = 47 for discovery cohort, *n* = 322 for replication cohort) and plasma samples (*n* = 48 for discovery cohort, *n* = 328 for replication cohort) was performed on a Bruker DRX600 spectrometer equipped with either a 5-mm TXI probe operating at 600.13 MHz or a 5-mm BBI probe operating at 600.44 MHz. The 90° pulse length was determined prior to each run and field frequency was locked using D_2_O as solvent. Detailed procedures are included in supplementary materials.

### Transcriptomics

Transcriptomic analyses from liver biopsies have been previously described [5] and detailed procedures are included in supplementary materials.

### Microbiome analyses

Bacterial population in mouse faeces from studies 1 and 3 was determined using next-generation high throughput sequencing of variable regions of the 16S rRNA bacterial gene, whereas a shotgun metagenomic sequencing was employed for mice study 2 and human cohorts. Detailed protocols are included in supplementary materials.

### Primary human hepatocytes culture and treatments

Cryopreserved primary human hepatocytes (HH) were commercially sourced (Innoprot, Bizkaia, Spain) and cultured with hepatocytes medium (Innoprot) supplemented with 5% fetal bovine serum, 1% hepatocytes growth supplement (mixture of growth factors, hormones and proteins necessary for culture of primary hepatocytes) and 100 U/ml penicillin and streptomycin. HH were grown on poly-l-lysine pre-coated cell dishes at 37 °C and 5% CO_2_ atmosphere following manufacturer’s recommendations. Cells were treated with iron for 48 or 72 h alone, or in combination with palmitic acid (PA) for 24 h following the iron 48/72 h treatment. Compounds were prepared as follows: 27.84 mg of PA (Sigma, San Luis, MO) was dissolved in 1 ml sterile water to obtain a 100 mM stock solution. Five percent bovine serum albumin (BSA) was prepared in serum-free DMEM and then mixed with PA stock solution for at least 1 h at 40 °C to obtain a 5 mM solution. Iron was dissolved in water. Iron was used at 50 μM for 48 or 72 h, and PA 200 μM for 24 h. BSA was used in all treatments as the vehicle. All experimental conditions were performed in 4 biological replicates. After treatment, cells were washed with PBS and collected with Qiazol for RNA purification or fixed with paraformaldehyde 4% for Oil Red O staining. After fixation, cells were dipped in isopropanol 60% before completely dried and stained with Oil Red O (Sigma, Lyon, France) for 10 min at room temperature. Pictures were taken using an inverted microscope.

### Gene expression analysis using real-time PCR (cells, mouse, human)

Total RNA was extracted and purified using RNeasy Mini Kit (QIAGEN, Gaithersburg, MD) following manufacturers’ protocol. Gene expression procedures were assessed using LightCycler 480 Real-Time PCR System (Roche Diagnostics SL, Barcelona, Spain), using Sybr-green technology suitable for relative genetic expression quantification. Peptidylprolyl isomerase A was used as housekeeping.

### Mice studies

Detailed procedure for the animal studies is included in supplementary materials. To assess the effects of dietary iron on the gut microbiome (mice study 1), 12-week-old male C57BL/6J mice were fed with low- (LI: 4 mg/kg), low-normal- (LNI: 35 mg/kg), high-normal- (HNI: 500 mg/kg), moderately high- (MHI: 2000 mg/kg) or high- (HI: 20,000 mg/kg) carbonyl iron diets for 9 weeks before sacrifice. The impact of different diets on the effects of dietary iron on the gut microbiome (mice study 2) was assessed by feeding mice with either a ‘no-High Fat Diet’ (No-HFD) or a ‘High Fat diet’ (HFD) with different concentrations of iron representing the LI, LNI, HNI and MHI diets. Finally, the causal role of the microbiome on iron status was evaluated by faecal microbiota transplantation from low- (*n* = 3) and high- (*n* = 3) ferritin donors matched for age and BMI to recipient mice (mice study 3).

### Statistical analysis

Ferritin distribution and normality was checked visually and using the Kolmogorov-Smirnov and Shapiro-Wilk tests and was found to be not normally distributed. Metagenomic, transcriptomic and metabolomic data were also not normally distributed. Therefore, all univariate correlations analyses were based on partial Spearman’s correlation (pSC) adjusting for age, BMI, sex and country. Multivariate analyses were performed on the log10 transformed data. Associations between serum ferritin and steatosis grade in the discovery and replication cohorts were assessed by non-parametric Kruskal Wallis test and steatosis grade 0 (absence) was compared to grades 1–3 using the non-parametric Wilcoxon-Mann-Whitney test. Associations between microbiome, transcriptomic or metabolomic data with serum ferritin quartiles were assessed by non-parametric Mann-Kendall test to detect monotonic trends. The Wilcoxon-Mann-Whitney test was used to individually compare the lowest ferritin quartile (Q1 used as reference) with the higher quartiles.

#### Microbiome data analysis (human)

Taxa were filtered so that only those having a relative abundance > 20% in at least 20% of the samples were considered for further analyses. To identify families and genera associated with serum ferritin, we applied both multivariate Mnet regularization regression [40] models and DESeq2 [41] analyses including age, BMI, country and hs-CRP as covariates using the R package ncvreg, phyloseq and DESeq2. The Mnet uses a combination of ridge (*l*_*2*_) and minimax concave penalties (MCP) to deal efficiently with *predictors* ≥ *n* problems with highly correlated predictors, which is typical of omics data. Combining both penalties, the Mnet performs variable selection by forcing the regression coefficients of variables not actually associated with the response to 0 and at the same time handles multicollinearity within the data. In the Mnet, there are three parameters that need to be tuned: the regularization parameter (λ ≥ 0) controlling the shrinkage of the variables, the elastic net mixing parameter (0 < α < 1) controlling the contribution of ridge (α = 0) and LASSO (α = 1) penalty to the model, and the MCP penalty (γ). These were optimized by 10-fold cross-validation. The relationship between serum ferritin quartiles and MGR was assessed using a generalized linear model (GLM) with a multinomial probability distribution and a cumulative logit as a link function to account for non-normality adjusting for age and BMI using SPSS.

#### Microbiome data analysis (mouse)

Alpha and beta diversity indices were obtained using the R package vegan. Trends between alpha diversity measures and iron doses for each diet were assessed by non-parametric Mann-Kendall test, whereas differences between HFD and no-HFD for each iron dose were assessed by non-parametric Wilcoxon-Mann-Whitney test. To estimate the relatedness of microbial communities among groups, beta diversity distances between samples were examined using principal coordinate analysis (PCoA). Differences in microbial composition were assessed by PERMANOVA analyses using the Adonis function in vegan R package with 999 permutations. Families and genera responsible for differences in the microbial compositions among iron doses for each diet were identified through multivariate O-PLS modelling, whereby the microbiome composition was used as the descriptor matrix (X) to predict the iron dose (Y). The predictive performance of the model (*Q*^2^*Y*) was calculated using a leave-one-out cross-validation approach and model validity was established by permutation testing (1000 permutations). Variable selection was based on O-PLS regression loadings adjusted for multiple testing using the Benjamini-Hochberg procedure (*p*FDR). A *p*FDR < 0.05 was used as the reference feature selection criterium.

#### Transcriptome and metabolome data analysis

For transcriptomic and metabolomic data, we used a combination of multivariate O-PLS modelling and partial Spearman’s correlation (pSC) using in-house MATLAB scripts. First, an O-PLS model was built. Here, the omics profiles were used as the descriptor matrix (X) to predict serum ferritin as the response variable (Y). Then, variable selection was achieved combining the variable importance for projection (O-PLS-VIP) [42] and the O-PLS regression loadings adjusted for multiple testing using the Benjamini-Hochberg procedure (*p*FDR). A *p*FDR < 0.05 was used as the reference feature selection criterium. However, a less restrictive threshold (*p*FDR < 0.1 unless otherwise indicated) was used to include variables with high VIP (> 1). Finally, each individual variable identified form multivariate models was further validated by pSC adjusting for age, BMI, sex and country.

#### Clustering analysis

Unsupervised hierarchical clustering analysis (HCA) was performed to identify general patterns of transcriptomic variation among serum ferritin quartiles. Significant transcripts associated with serum ferritin were used for sample clustering. Before clustering, data were standardized as *z* scores across samples for each transcript. This standardized matrix was then used in unsupervised HCA using Euclidean distances and Ward linkage. Heatmaps and dendrograms following HCA were generated using the heatmap.2 function from the gplots R package. In the heatmaps, a red-blue colour scale was used whereby shades of red and blue represent higher and lower values, respectively, compared with the mean.

#### Integration of datasets

To explore the functional associations among the microbiome changes and metabolic and transcriptomic perturbations associated with serum ferritin, were used two approaches. First, datasets were integrated using an O2-PLS approach. It is a bidirectional multivariate regression method that allows separate joint covariance between two blocks from systemic variation specific (orthogonal) to each block (X and Y) [43]. The number of components for the predictive and orthogonal blocks was selected based on a 7-fold cross-validation using the R package OmicsPLS [44]. Variable loadings for each block were scaled as correlation coefficient (pcorr and qcorr for X and Y, respectively) and represented in a correlation circle plot. Scaling was performed by calculating the correlation between each variable and its associated component. The longer the distance to the origin, the stronger the relationship between variables. Strongly positively associated variables or groups of variables are projected closely to each other on the correlation circle (~ 0° angle). The variables or groups of variables strongly negatively associated are projected diametrically opposite (~ 180° angle) on the correlation circle. Variables not correlated are situated ~ 90° one from the other. Additionally, univariate partial Spearman’s correlations adjusted for age, BMI, sex and country were calculated and represented as correlation heatmaps. Only significant correlations (*p* < 0.05) are displayed. A pFDR correction was used to adjust *P* values for multiple testing. Significant associations after a pFDR correction (< 0.05) are highlighted with a black box.

#### Pathway analysis

Differentially expressed genes associated with serum ferritin were annotated via over-representation analysis using the Consensus Pathway database (CPDB) [45]. Pathway significance was assessed using a hypergeometric test and a Bonferroni procedure was applied for multiple testing correction.

## Supplementary Information

**Additional file 1:.** Baseline characteristics of the independent cohort.

**Additional file 2:.** Gut microbiome bacterial species associated with the serum ferritin. Results of differential bacterial abundance associated with ferritin as calculated from shotgun metagenomic sequencing in an independent cohort of obese and non-obese subjects, adjusting for age, BMI, sex, and hs-CRP.

**Additional file 3:.** Metagenome functions based on KEGG annotation associated with the serum ferritin. Results of metagenome KEGG functions associated with ferritin as calculated from shotgun metagenomic sequencing in an independent cohort of obese and non-obese subjects, adjusting for age, BMI, sex, and hs-CRP.

**Additional file 4:.** Metagenome functions based on EggNOG annotation associated with the serum ferritin. Results of metagenome EggNOG functions associated with ferritin as calculated from shotgun metagenomic sequencing in an independent cohort of obese and non-obese subjects, adjusting for age, BMI, sex, and hs-CRP.

**Additional file 5: Supplementary methods**. Methods for the animals studies, NMR metabolomics, liver transcriptomics, and 16S rRNA and shotgun metagenomics sequencing.

**Additional file 6: Figure S1**. Flow chart of the study human cohorts and omics analyses pipeline.

**Additional file 7: Figure S2**. Associations of serum ferritin with hs-CRP. a) Association of hs-CRP with serum ferritin quartiles in the discovery cohort and b) an independent cohort of obese and non-obese patients (Mann-Kendall trend test and Wilcoxon tests).

**Additional file 8: Figure S3**. Associations of serum ferritin with the gut microbiome in the human cohorts. Permutation tests for the goodness-of-fit (*R*^2^*Y*) and goodness of prediction (*Q*^2^*Y*) obtained from the O-PLS model between serum ferritin and a) bacterial families or b) bacterial genera in a subsample of obese women from the discovery and replication cohorts from Italy and Spain (*n* = 56). c) Significant families and d) genera associated with serum ferritin from O-PLS regression loadings. Families and genera associated positively and negatively associated with serum ferritin from Mnet regression models are highlighted in dark red and blue, respectively. e) Significant families and f) genera associated with serum ferritin after further validation of the O-PLS significant variables by pSC adjusting for age, BMI, country, and hs-CRP. g) Associations of bacterial families and h) genera associated with serum ferritin by DESeq2 analysis from shotgun metagenomic sequencing data in the independent cohort of obese and non-obese patients (*n* = 130), adjusting for age, BMI, sex, and hs-CRP. Families and genera also associated with serum ferritin in the discovery and replication cohorts based on Mnet regression models are highlighted in dark red, whereas those also identified from O-PLS modelling are highlighted in dark pink.

**Additional file 9: Figure S4**. Associations of bacterial families with iron-related genes (discovery cohort, *n* = 35). Only significant correlations are coloured. Genes were measured by real time-PCR. Bacterial families with a significant positive association with serum ferritin concentrations are highlighted in dark red, whereas those with a significant negative association are highlighted in dark blue. *ChREBP*, carbohydrate response element binding protein; *LCN2*, Lipocailin 2; *MitoNEET*, Mitochondrial Inner NEET Protein; *TFRC,* Transferrin Receptor; SLC40A1, Solute Carrier Family 40 Member 1 (Ferroportin); *TF*, Transferrin; *FTH1*, Ferritin Heavy Chain 1; HAMP, Hepcidin Antimicrobial Peptide; *FTL*, Ferritin Light Chain; *IRP1*, Iron Regulatory Protein 1.

**Additional file 10: Figure S5**. Associations the gut microbiome composition with hs-CRP and the gut microbiome functionality with serum ferritin. a) Volcano plot of differential bacterial genera and b) taxa associated with hs-CRP as calculated by DESeq2 from shotgun metagenomic sequencing in the independent cohort of obese and non-obese patients, adjusting for age, BMI, and sex. Fold change associated with a unit change in hs-CRP and adjusted *p*-values are plotted for each genus or taxon, respectively. Significantly different taxa are coloured according to phylum. c) Permutation test for the goodness-of-fit (*R*^2^*Y*) and goodness of prediction (*Q*^2^*Y*) obtained from the O-PLS model between serum ferritin and metagenome functions in the independent cohort (*n* = 130 obese and non-obese patients). d) Significant metagenome functions based on EggNOG functional annotations associated with serum ferritin in the independent cohort (*n* = 130 obese and non-obese patients). Initially, a significant O-PLS model between serum ferritin and metagenome functions was obtained for the independent cohort of obese and non-obese patients (*R*^*2*^*Y*=0.69, *Q*^*2*^*Y*=0.36, *p*<0.001). Then, significant O-PLS variables were further validated by pSC adjusting for age, sex, and BMI.

**Additional file 11: Figure S6.** Associations of ferritin-related transcripts with liver fat accumulation. a) Permutation tests for the goodness-of-fit (*R*^*2*^*Y*) and goodness of prediction (*Q*^*2*^*Y*) obtained from the O-PLS model between the liver fat accumulation degree and transcripts that were significantly associated with serum ferritin. b) Significant transcripts associated with liver fat accumulation from O-PLS regression loadings. Hepatic genes belonging to the transcriptomic signature associated with serum ferritin and the gut microbiome are highlighted in dark red and blue. c) Further validation of O-PLS identified transcripts by pSC adjusting for age, BMI, sex, and country. d-g) Boxplots showing four hepatic genes identified in the transcriptomic signature associated with serum ferritin and the microbiome according to the serum ferritin quartiles (*Q*1-*Q*4).

**Additional file 12: Figure S7.** Iron supplementation leads to triglyceride accumulation and induces the expression of lipid and iron metabolism genes in primary human hepatocytes. a) Micrographs of primary human hepatocytes stained with Oil Red-O (representative images are from *n* = 4 independent batches). b) Quantification of lipid accumulation. O.D., Optical Density. c-h) *FABP4, FABP5, FATP5, CD36*, *FTH,* and *FTL* expression in hepatocytes. Data are mean ± SEM. Comparisons by one-way ANOVA. *p<0.05, **p<0.01, ***p<0.001 compared to control group based on *t*-test. ^#^*p*<0.05, ^##^*p*<0.01, ^###^*p*<0.001 compared to PA group based on *t*-test. Ctrl, control group; PA, palmitic acid; Fe48h, pre-treatment iron 50μM for 48h; Fe72h, pre-treatment iron 50μM for 72h; Fe48h + PA, pre-treatment iron 50μM for 48h + palmitic acid 200μM for 24h; Fe72h + PA, pre-treatment iron 50μM for 72h + palmitic acid 200μM for 24h.

**Additional file 13: Figure S8.** PcoA based on Canberra beta diversity comparing high fat diet (HFD) and non-high fat diet (No-HFD) for different iron doses. a) low-iron (LI) fed mice, b) low-normal-iron (LNI) fed mice, c) the high-normal iron (HNI) fed mice, d) moderately-high (MHI) iron fed mice. Differences in microbial composition were assessed by PERMANOVA analyses using the Adonis function in vegan R package with 999 permutations.

## Data Availability

The datasets that support the findings of the study are available from the corresponding author upon reasonable request (jmfreal@idibgi.org). The raw metagenomic sequence data for the FLORINASH cohort (with human-associated reads removed) have been deposited under the study accession number PRJEB14215 (secondary accession number ERP015847). The raw 16S rRNA gene sequence data associated with the mouse FMT work have been deposited under the study accession number PRJEB24891.
