# Supplementary material for: Iron status influences non-alcoholic fatty liver disease in obesity through the gut microbiome
Source: Microbiome. 2021 May 7;9:104. doi: 10.1186/s40168-021-01052-7 (PMC8106161; doi:10.1186/s40168-021-01052-7)
Supplement: Supplementary file 6 — Additional file 5: Supplementary methods. Methods for the animals studies, NMR metabolomics, liver transcriptomics, and 16S rRNA and shotgun metagenomics sequencing. [file 40168_2021_1052_MOESM6_ESM.docx]

**Supplementary Methods**

**Dietary iron in mice (study 1).** Twelve week-old male C57BL/6J mice were fed with low- (LI: 4 mg/kg TD. 10210, Harland, Teklad), low-normal- (LNI: 35 mg/kg, TD.10211, Harlan Teklad), high-normal- (HNI: 500 mg/kg, TD.10212, Harlan Teklad), moderately high- (MHI: 2000 mg/kg, TD.10214, Harlan Teklad), or high- (HI: 20000 mg/kg) carbonyl iron diets for 9 weeks before sacrifice. The LNI diet is derived from the AIN93G diet recommended by the American Institute of Nutrition, the HNI diet is based on animal facility diets, the MHI diet results in a modest two-fold increase in liver iron, which is within the 4-fold range observed in normal humans without iron-related pathology (1), and the HI diet was used to mimic hemochromatosis. Diets contained 60% carbohydrate, 17.7% protein, and 7.2% fat by weight. At the end of the study, pellets were freshly collected directly from the anus of mice, and immediately flash frozen in liquid Nitrogen. Samples were stored at -80ºC until analysis. Procedures were approved by the Institutional Animal Care and Use Committee of the University of Utah. Experimental groups were randomly allocated.

**Dietary iron impact in high fat diet in mice (study 2).** Adult male mice from strain C57BL/6 were bred in the rodent breeding facilities of the Wake Forest® School of Medicine. At 16-weeks of age, selected mice were separated in groups and started their diet treatments, which lasted for ten weeks. Two diets were administered to mice. The first diet called 'no-High Fat Diet' (No-HFD) was characterized by a supply of 17% energy as fat and regular drinking water. The second diet, called 'High Fat Diet' (HFD) consisted of a 42% energy as fat (12% saturated fat and 0.2% cholesterol) in chow with glucose (18.9 g/L) and fructose (23.1 g/L) in their drinking water. For each diet, four subgroups of 5 to 6 mice were feed with different concentrations of iron representing the LI, LNI, HNI, and MHI diets (4, 35, 500 and 2000 mg iron/kg chow, respectively). At the age of 26 weeks old, pellets were freshly collected directly from the anus of mice, and immediately flash frozen in liquid Nitrogen. Samples were stored at -80ºC until analysis. The mouse experiments were not performed in a blind manner. Experimental groups were randomly allocated.

**Faecal microbiota transplantation in mice (study 3).** Faecal microbiota transplantation experiments were performed previously and results have already been published (2). Briefly, faecal samples from low- (*n* = 3) and high- (*n* = 3) ferritin donors matched for age and BMI were suspended in sterile reduced PBS. Eight mice (8-week old C57BL6 male, Charles River) per patient were treated with an antibiotic mixture for 7 days and after a 4-day washout, mice were administered 20mg/day of faecal matter for 4 consecutive days. Two weeks later, mice were sacrificed and liver and plasma collected and frozen. Experimental groups were randomly allocated. All animal experimental procedures were approved by the local ethical committee (approval number 31-278) of Rangueil University Hospital (Toulouse, France).

**^1^H nuclear magnetic resonance spectroscopy-based metabolic profiling.** Spectroscopic analysis of urine (*n* = 47 for discovery cohort, *n* = 322 for replication cohort) and plasma samples (*n* = 48 for discovery cohort, *n* = 328 for replication cohort) was performed on a Bruker DRX600 spectrometer equipped with either a 5-mm TXI probe operating at 600.13 MHz or a 5-mm BBI probe operating at 600.44 MHz. The 90º pulse length was determined prior to each run and field frequency was locked using D_2_O as solvent.

*Urine samples.* All urine samples were thawed at room temperature and briefly centrifuged. Then, 350 µL urine were aliquoted into 5-mm NMR tubes, combined with 150 µL phosphate buffer (pH 7.4; 90%D_2_O:10%H_2_O) containing 1 mM of the internal standard 3-trimethylsilylpropionate (TSP) and sodium azide, and the mixture was briefly vortexed. A standard one-dimensional (1-D) NOESY presaturation pulse sequence (RD-90º-*t_1_*-90º-*t_m_*-90º-acquire) using the Bruker program *noesypr1d*. Water suppression was achieved by noise irradiation using a recycle delay (RD) of 2 s. For each sample, 128 scans were collected into 32K data points with a spectral width of 20 ppm.

*Plasma samples.* Plasma samples were thawed at room temperature and 350 µL aliquots were carefully placed in 5-mm NMR tubes. Then, 150 µL of saline solution (0.9% NaCl prepared with 80:20 H_2_O/D_2_O and sodium azide) were added and the mixture was gently vortexed. Spectra were acquired using a water suppressed Carr-Purcell-Meiboom-Gill (CPMG) using the Bruker program cpmgpr (RD [90º-(τ-180º-τ) n-acquire). A RD of 2 s was employed for net magnetization relaxation, during which noise irradiation was applied in order to suppress the large water proton signal. A number of loops *n* = 100 and a spin-echo delay τ = 400 µs was used to allow spectral editing through T2 relaxation and therefore attenuation of broad signals. For each sample, 128 scans were recorded in 32K data points with a spectral with of 20 ppm.

*NMR data processing of urine and plasma samples.* All NMR spectra were processed using Topspin (Bruker Biospin, UK). Free induction decays (FID) were multiplied by an exponential function corresponding to a line broadening of 0.3 Hz prior to the Fourier transformation. Spectra were automatically phased, baseline corrected and referenced to TSP (0.0 ppm) for urine and the center of the anomeric doublet of glucose (5.23 ppm) for plasma samples. Baseline and peak alignment quality control was done by individual verification for each spectrum, and occasionally a spectrum was manually adjusted. Spectral line-shape quality was also individually assessed, and occasionally spectra were re-acquired during the same sample run. The spectra were all then imported to MATLAB and the region around the water resonance (δ = 4.70-4.90 for urine and δ = 4.5-5.0 for plasma) was removed. All NMR spectra were normalized using a probabilistic quotient algorithm (3).

**Transcriptomics.** Transcriptomic analyses have been previously described (2). Briefly, RNA from liver biopsy samples was extracted using standard extraction protocols (TRIzol) by Miltenyi Biotec as previously reported. RNA quality (gel images, RNA integrity number and electropherograms) was assessed using an Agilent 2100 Bioanalyzer platform (Agilent Technologies). An RNA integrity number > 6 was considered sufficient for gene expression experiments (4). One-hundred ng of total RNA was used for linear T7-based amplification of RNA for each sample. cRNA was prepared by amplification of the RNA and labelled with Cy3 using the Agilent Low Input Quick Amp Labeling Kit according to the manufacturer’s instructions. The amounts of cRNA and dye that were incorporated were measured by an ND-1000 spectrophotometer (NanoDrop Technologies). Hybridization of the Agilent Whole Human Genome Oligo Microarrays 4 × 44K was done following the Agilent 60-mer oligo microarray processing protocol using the Agilent Gene Expression Hybridization Kit. The fluorescence signals of the hybridized Agilent microarrays were detected using Agilent’s Microarray Scanner after washing with Agilent Gene Expression Wash Buffer twice and with acetonitrile once. Feature intensities were determined using Agilent Feature Extraction Software.

Microarray data were processed and normalized using R and the BioConductor package LIMMA (Linear Models for Microarray Data) (5). Raw data quality was assessed using pseudoMA and box plots. A background correction was applied and normalization of the green channel between arrays was done using ‘cyclicloess’ between pairs of arrays. Control and low-expressed probes were removed and only those probes brighter than the negative controls (≥ 10%) on at least one array were kept. Batch-corrected data was obtained using removeBatchEffect based on ‘Batch’ (5). Probes with no associated gene ID were removed. Finally, data were averaged based on an association to a particular gene.

**16S rRNA gene sequencing (mouse studies 1 and 3).** Bacterial population in mouse faeces from studies 1 and 3 was determined using next generation high throughput sequencing of variable regions of the 16S rRNA bacterial gene by Vaiomer (Vaiomer SAS, Labège, France) as previously described. Briefly, total DNA was extracted using the QIAamp DNA Stool Mini Kit (Qiagen) after two mechanical lysis steps in a bead beater (TissueLyser; Qiagen). Quality and quantity of extracted DNA were evaluated by gel electrophoresis and ND-2000 spectrophotometer (6).

*Library construction and sequencing*. PCR amplification was performed using 16S universal primers targeting the V3‐V4 region of the bacterial 16S ribosomal RNA gene (Vaiomer universal 16S primers) as previously described (6). The joint pair length was set to encompass 476 base pairs amplicon thanks to 2 *x* 300 paired‐end Miseq kit V3. For each sample, a sequencing library was generated by addition of sequencing adapters. The detection of the sequencing fragments was performed using MiSeq Illumina® technology.

*Bioinformatics pipeline.* The targeted 16S sequences from stool microbiota were analysed using the bioinformatics pipeline established by Vaiomer using FROGS (7). Briefly, after demultiplexing of the bar-coded Illumina paired reads, single read sequences were cleaned and paired for each sample independently into longer fragments. After quality filtering and alignment against a 16S reference database, a clustering into OTU (Operational Taxonomic Unit) with a 97% identity threshold, and a taxonomic assignment were performed in order to determine community profiles.

**Metagenomics (mouse study 2 and human cohorts of obese and non-obese patients).** Faecal pellets were sent to FISABIO facilities (Valencia, Spain) for total DNA extraction and sequencing. Bacterial DNA was isolated following the QIAamp Fast DNA Stool Mini kit (Qiagen, Valencia, CA, USA) with slight modifications. For each sample, two frozen pellets were transferred into sterile 2 ml microcentrifuge tubes and partial homogenization was achieved by manual disruption with 200 µl sterile barrier tips (Bio Pointe Scientific Inc, Mexico) in 1 mL InhibiteEX Buffer. After heating the homogenates at 95ºC for 5 min, an additional vortex for 10 min at maximum setting with 250 mL of Zirconia Beads (Ambion Inc, Austin, TX, USA) was added in order to ensure an optimized bacterial cell wall disruption. For the rest of the DNA isolation protocol the manufacturer’s instructions were followed. Two additional extractions were carried out together with the samples to serve as extraction negative controls. After preparation, DNAs were quantified using a Qubit® 3.0 Fluorometer (Thermo Fisher Scientific, Carlsbad, CA, USA), and shot gun libraries were prepared for high-throughput sequencing using the Nextera XT DNA Library Prep kit (Illumina, CA, USA) according to the manufacturers’ protocol. Sequencing was carried out by MiSeq Paired End Illumina Technology, using the Reagent Kit V3 for 300 bp Paired-End reads.

Bioinformatic pre-processing of the bacterial metagenomes was carried out as follows. Raw sequence reads were first assessed for the presence of partial adapter sequences at the 3’ end and, when identified, those adapters were removed, using a combination of in-house shell commands and CutAdapt (8). Next, reads were quality and length- filtered using Prinseq-lite (v0.20.4) (9), by first trimming the ends with quality (*Q*) < 30, and removing reads shorter than 100 bp, as well as those containing poli-tails, Ns and low complexity sequences. In a second step, reads showing low average quality (*Q* < 20) after trimming the ends were concordantly removed. The R1 and R2 reads were concordantly joined with COPE (10), remaining unjoined reads in separate files from the joined ones. Next, a deduplication of the filtered reads with prinseq-lite-0.20.4 allowed the removal of identical reads in length and sequence. After that, FASTQ files were converted into FASTA format, and those files were clusterized using USEARCH v8.1.1861 at 99% identity (11), in a single file for joined reads and another file for unjoined ones. Both files were separately mapped against the mouse genome using Bowtie2 (12), with end-to-end and very sensitive options, to remove host reads. The resulting files were the input for a BLASTn search (e-value <10^-3^) against a custom-made bacterial database, based on updated to June 2016 (identity ≥ 80% along ≥ 75% of the read length). Using the map of clusters generated by USEARCH, those reads with BLASTn assigned taxonomy were split into individual samples. An in-house script was then used to compare the identity and alignment length of the different potential hits matching for each query and an LCA strategy was used to assign the best hit when possible to the closest-to-the–tip taxonomic level, or an undetermined hit if not possible. In the latter, undetermined assignments were indicated by the addition of a ‘_u’ to the end of the name of the last unambiguous taxonomic level and repeating this process for each undetermined taxonomic rank as descending to the species level. Finally, the list of the resulting hits was counted for each sample and a series of operational taxonomic units (OTU) tables were constructed. Since the number of bacterial hits was variable among samples, a sub-sampling comprising the number of reads of the sample accounting for the fewest reads (S02; *n* = 31432 reads) was carried out on all samples. Therefore, for downstream analyses, all samples contained the same number of reads.

**Metagenomics (discovery and validation cohorts).** Shotgun sequencing data were generated for *n* = 56 obese women and have been previously reported (2). Briefly, faecal total DNA was isolated using the QIAamp DNA Stool Mini kit (Qiagen), with slight modifications by adding a bead-beating step. Raw sequence reads were assessed for the presence of adapter sequences at the 3’ end. Next, reads were quality- and length- filtered using Trim Galore by discarding the ends with low quality (*Q* < 20) and removing reads < 50 nucleotides. Reads were binned to higher taxa by alignment to reference databases using the BWA MEM algorithm. The taxonomic composition of each sample was identified using MetaPhlAn2.0 (13). Metagenome assembly was performed using IDBA-UD (14). Initially, an independent assembly was carried out for each sample. In a second round, unassembled reads were pooled and assembled to improve the representation of low-abundance sequences. Gene prediction was carried out using MetaGeneMark (15) and translated into protein sequences, which were clustered using UCLUST (11) at 95% identity. A non-redundant gene catalog was formed based on centroid sequences for each cluster, which was used to determine the gene abundance in each sample by aligning the reads against the gene catalog using BWA MEM (16).

**Microbial gene richness (MGR).** MGR results for the 56 patients for whom metagenomic data were available have been previously reported (2). In brief, metagenomic data were adjusted for technical variability and sequencing depth by random selection of 7 million reads mapped to the gene catalog for each sample. Then, the mean number of genes over 30 random drawing was computed.

**SI References**

1. Milman N, Graudal N, Hegnhoj J, Christoffersen P, Strandberg Pedersen N. Relationships among serum iron status markers, chemical and histochemical liver iron content 117 patients with alcoholic and non-alcoholic hepatic disease. Hepatogastroenterology. 1994;41:20–24.

2. Hoyles L, Fernández-Real J-M, Federici M, Serino M, Abbott J, Charpentier J, et al. Molecular phenomics and metagenomics of hepatic steatosis in non-diabetic obese women. Nat. Med. 2018;24:1070–1080.

3. Dieterle F, Ross A, Schlotterbeck G, Senn H. Probabilistic quotient normalization as robust method to account for dilution of complex biological mixtures. Application in 1H NMR metabonomics. Anal. Chem. 2006;78:4281–4290.

4. Fleige S, Pfaffl MW. RNA integrity and the effect on the real-time qRT-PCR performance. Mol. Aspects Med. 2006;27:126–139.

5. Smyth GK. limma: Linear Models for Microarray Data. In: Bioinformatics and Computational Biology Solutions Using R and Bioconductor. New York: Springer-Verlag; 2005. p. 397–420.

6. Lluch J, Servant F, Païssé S, Valle C, Valière S, Kuchly C, et al. The Characterization of Novel Tissue Microbiota Using an Optimized 16S Metagenomic Sequencing Pipeline. PLoS One. 2015;10:e0142334.

7. Escudié F, Auer L, Bernard M, Mariadassou M, Cauquil L, Vidal K, et al. FROGS: Find, Rapidly, OTUs with Galaxy Solution. Bioinformatics. 2018;34:1287–1294.

8. Martin M. Cutadapt removes adapter sequences from high-throughput sequencing reads. EMBnet.journal. 2011;17:10.

9. Schmieder R, Edwards R. Quality control and preprocessing of metagenomic datasets. Bioinformatics. 2011;27:863–864.

10. Liu B, Yuan J, Yiu S-M, Li Z, Xie Y, Chen Y, et al. COPE: an accurate k-mer-based pair-end reads connection tool to facilitate genome assembly. Bioinformatics. 2012;28:2870–4.

11. Edgar RC. Search and clustering orders of magnitude faster than BLAST. Bioinformatics. 2010;26:2460–2461.

12. Langmead B, Salzberg SL. Fast gapped-read alignment with Bowtie 2. Nat. Methods. 2012;9:357–9.

13. Truong DT, Franzosa EA, Tickle TL, Scholz M, Weingart G, Pasolli E, et al. MetaPhlAn2 for enhanced metagenomic taxonomic profiling. Nat. Methods. 2015;12:902–3.

14. Peng Y, Leung HCM, Yiu SM, Chin FYL. IDBA-UD: a de novo assembler for single-cell and metagenomic sequencing data with highly uneven depth. Bioinformatics. 2012;28:1420–8.

15. Zhu W, Lomsadze A, Borodovsky M. Ab initio gene identification in metagenomic sequences. Nucleic Acids Res. 2010;38:e132–e132.

16. Le Chatelier E, Nielsen T, Qin J, Prifti E, Hildebrand F, Falony G, et al. Richness of human gut microbiome correlates with metabolic markers. Nature. 2013;500:541–546.
