# Supplementary figures and images for: Iron status influences non-alcoholic fatty liver disease in obesity through the gut microbiome
Source: Microbiome. 2021 May 7;9:104. doi: 10.1186/s40168-021-01052-7 (PMC8106161; doi:10.1186/s40168-021-01052-7)

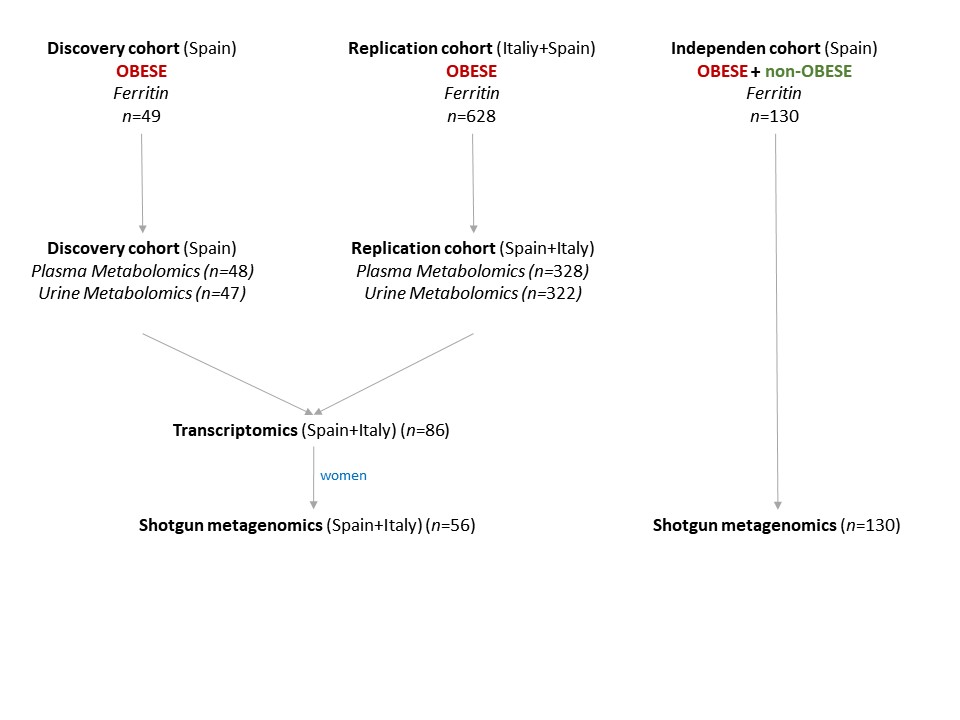

Supplement: Supplementary file 7 — Additional file 6: Figure S1. Flow chart of the study human cohorts and omics analyses pipeline. [file 40168_2021_1052_MOESM7_ESM.jpg]

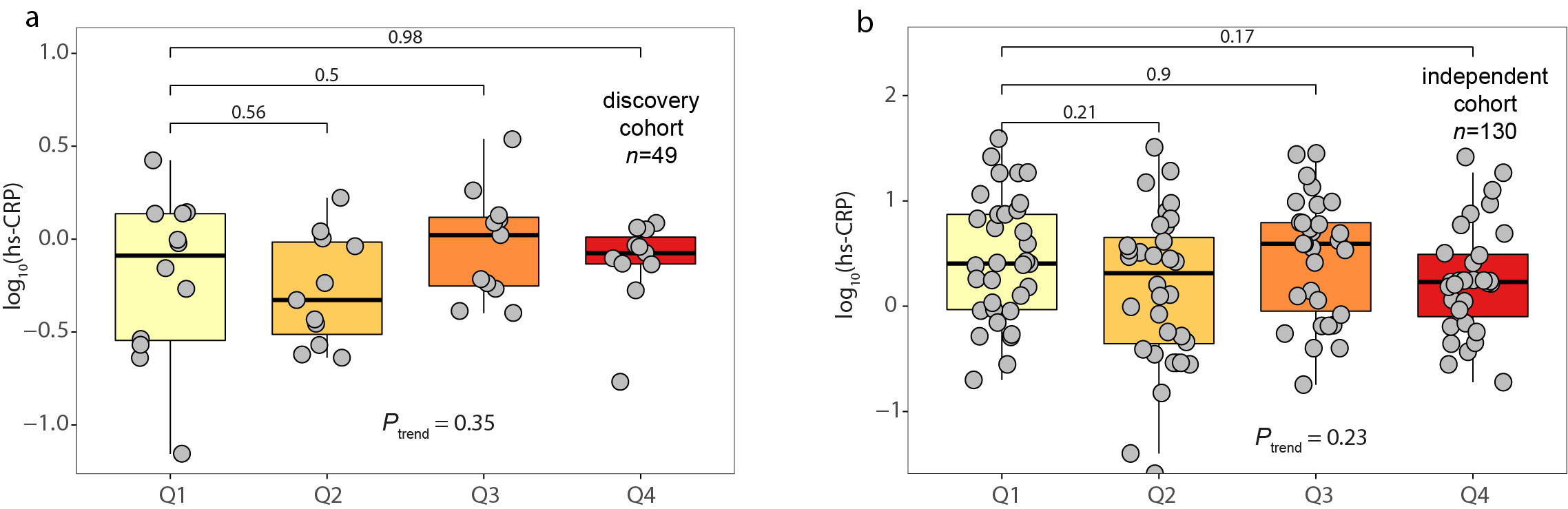

Supplement: Supplementary file 8 — Additional file 7: Figure S2. Associations of serum ferritin with hs-CRP. a) Association of hs-CRP with serum ferritin quartiles in the discovery cohort and b) an independent cohort of obese and non-obese patients (Mann-Kendall trend test and Wilcoxon tests). [file 40168_2021_1052_MOESM8_ESM.jpg]

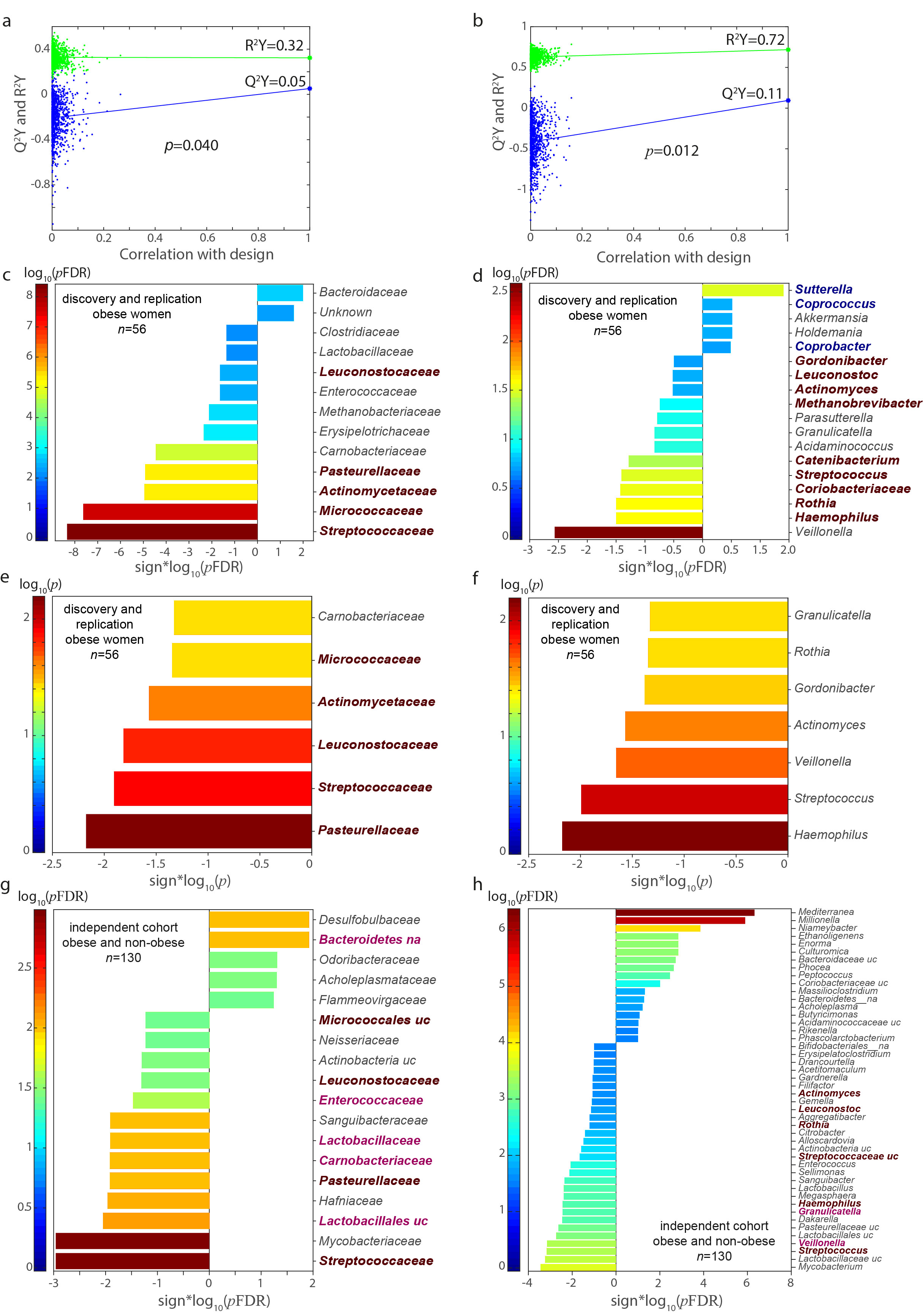

Supplement: Supplementary file 9 — Additional file 8: Figure S3. Associations of serum ferritin with the gut microbiome in the human cohorts. Permutation tests for the goodness-of-fit (R2Y) and goodness of prediction (Q2Y) obtained from the O-PLS model between serum ferritin and a) bacterial families or b) bacterial genera in a subsample of obese women from the discovery and replication cohorts from Italy and Spain (n = 56). c) Significant families and d) genera associated with serum ferritin from O-PLS regression loadings. Families and genera associated positively and negatively associated with serum ferritin from Mnet regression models are highlighted in dark red and blue, respectively. e) Significant families and f) genera associated with serum ferritin after further validation of the O-PLS significant variables by pSC adjusting for age, BMI, country, and hs-CRP. g) Associations of bacterial families and h) genera associated with serum ferritin by DESeq2 analysis from shotgun metagenomic sequencing data in the independent cohort of obese and non-obese patients (n = 130), adjusting for age, BMI, sex, and hs-CRP. Families and genera also associated with serum ferritin in the discovery and replication cohorts based on Mnet regression models are highlighted in dark red, whereas those also identified from O-PLS modelling are highlighted in dark pink. [file 40168_2021_1052_MOESM9_ESM.jpg]

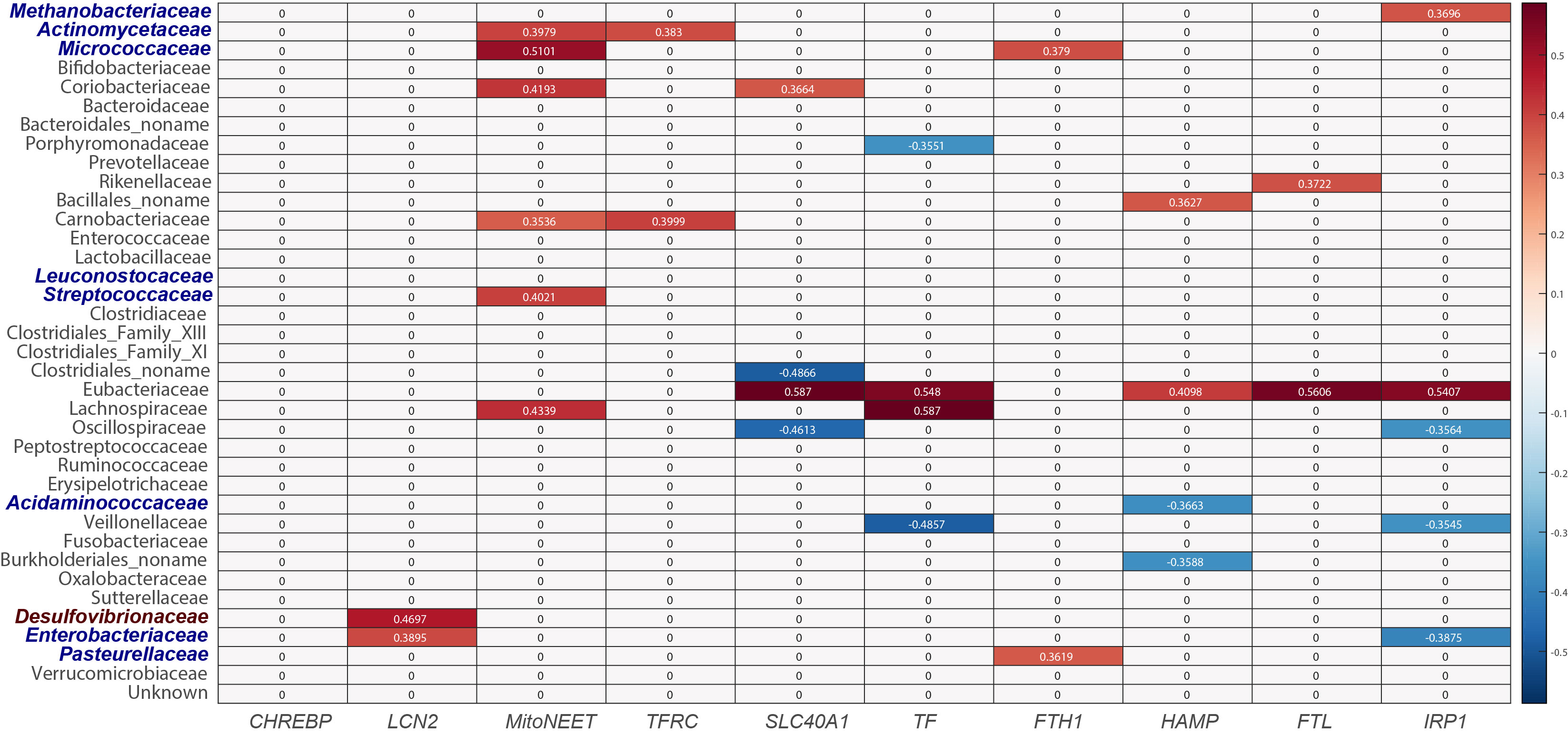

Supplement: Supplementary file 10 — Additional file 9: Figure S4. Associations of bacterial families with iron-related genes (discovery cohort, n = 35). Only significant correlations are coloured. Genes were measured by real time-PCR. Bacterial families with a significant positive association with serum ferritin concentrations are highlighted in dark red, whereas those with a significant negative association are highlighted in dark blue. ChREBP, carbohydrate response element binding protein; LCN2, Lipocailin 2; MitoNEET, Mitochondrial Inner NEET Protein; TFRC, Transferrin Receptor; SLC40A1, Solute Carrier Family 40 Member 1 (Ferroportin); TF, Transferrin; FTH1, Ferritin Heavy Chain 1; HAMP, Hepcidin Antimicrobial Peptide; FTL, Ferritin Light Chain; IRP1, Iron Regulatory Protein 1. [file 40168_2021_1052_MOESM10_ESM.jpg]

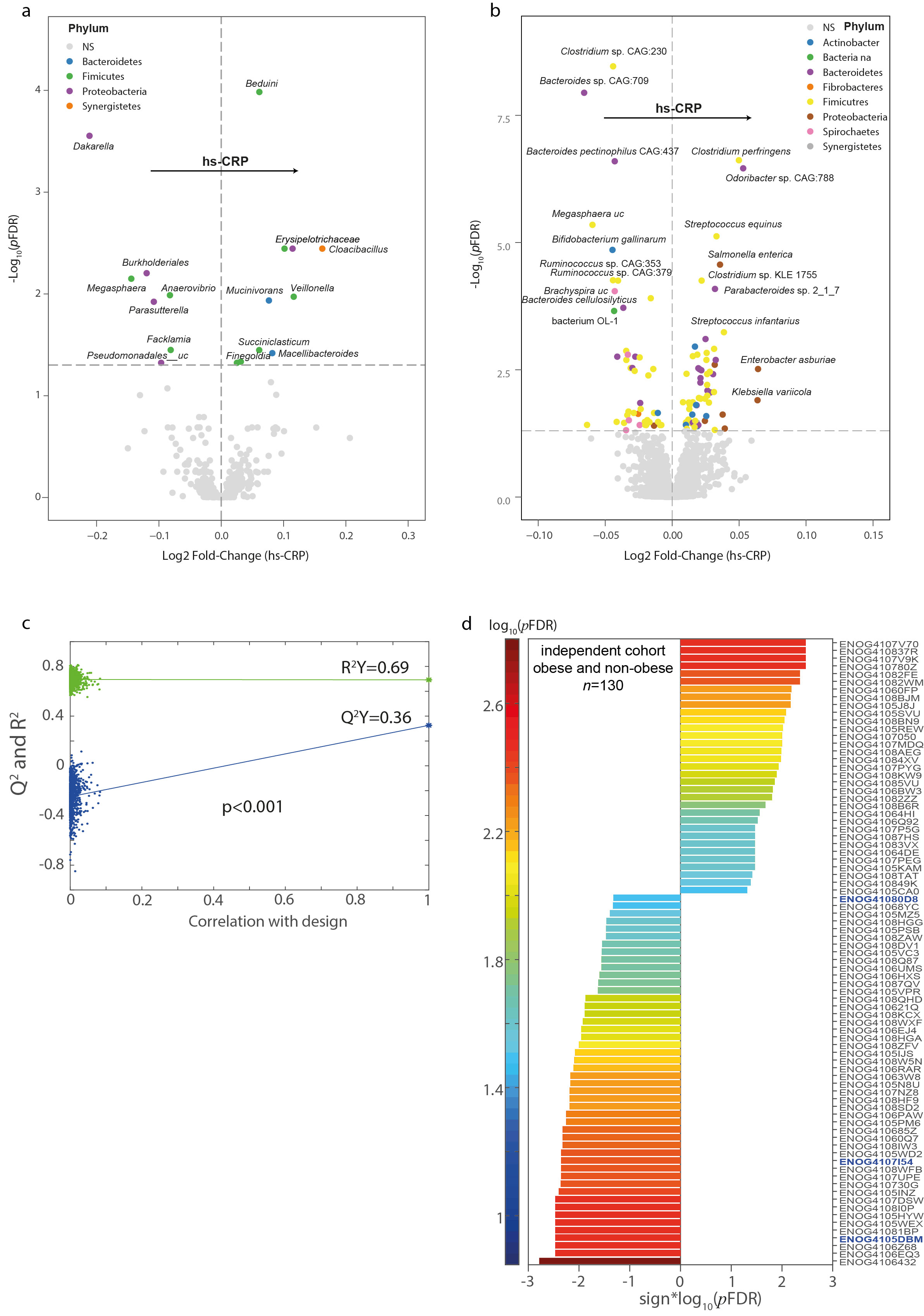

Supplement: Supplementary file 11 — Additional file 10: Figure S5. Associations the gut microbiome composition with hs-CRP and the gut microbiome functionality with serum ferritin. a) Volcano plot of differential bacterial genera and b) taxa associated with hs-CRP as calculated by DESeq2 from shotgun metagenomic sequencing in the independent cohort of obese and non-obese patients, adjusting for age, BMI, and sex. Fold change associated with a unit change in hs-CRP and adjusted p-values are plotted for each genus or taxon, respectively. Significantly different taxa are coloured according to phylum. c) Permutation test for the goodness-of-fit (R2Y) and goodness of prediction (Q2Y) obtained from the O-PLS model between serum ferritin and metagenome functions in the independent cohort (n = 130 obese and non-obese patients). d) Significant metagenome functions based on EggNOG functional annotations associated with serum ferritin in the independent cohort (n = 130 obese and non-obese patients). Initially, a significant O-PLS model between serum ferritin and metagenome functions was obtained for the independent cohort of obese and non-obese patients (R2Y=0.69, Q2Y=0.36, p<0.001). Then, significant O-PLS variables were further validated by pSC adjusting for age, sex, and BMI. [file 40168_2021_1052_MOESM11_ESM.jpg]

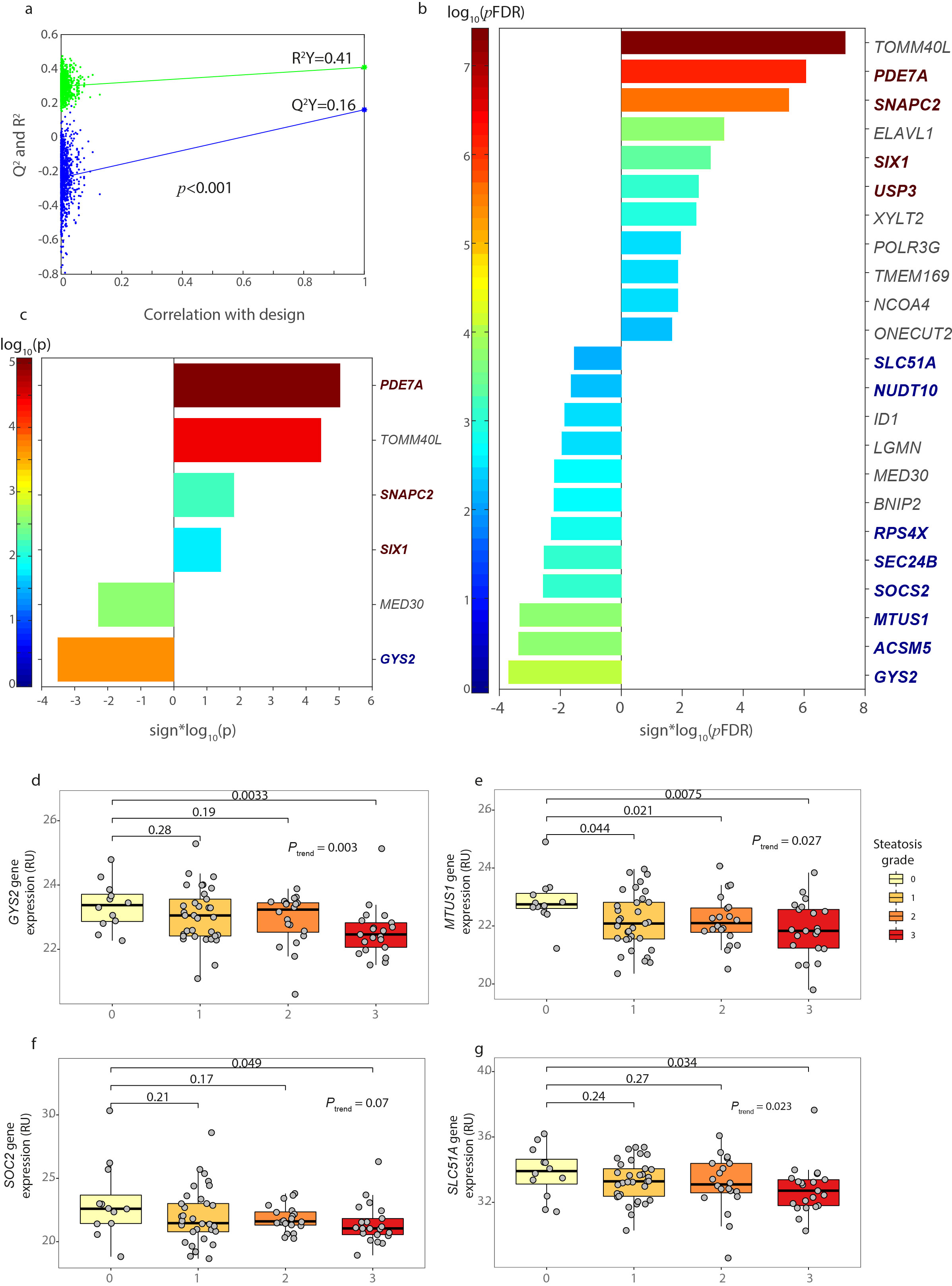

Supplement: Supplementary file 12 — Additional file 11: Figure S6. Associations of ferritin-related transcripts with liver fat accumulation. a) Permutation tests for the goodness-of-fit (R2Y) and goodness of prediction (Q2Y) obtained from the O-PLS model between the liver fat accumulation degree and transcripts that were significantly associated with serum ferritin. b) Significant transcripts associated with liver fat accumulation from O-PLS regression loadings. Hepatic genes belonging to the transcriptomic signature associated with serum ferritin and the gut microbiome are highlighted in dark red and blue. c) Further validation of O-PLS identified transcripts by pSC adjusting for age, BMI, sex, and country. d-g) Boxplots showing four hepatic genes identified in the transcriptomic signature associated with serum ferritin and the microbiome according to the serum ferritin quartiles (Q1-Q4). [file 40168_2021_1052_MOESM12_ESM.jpg]

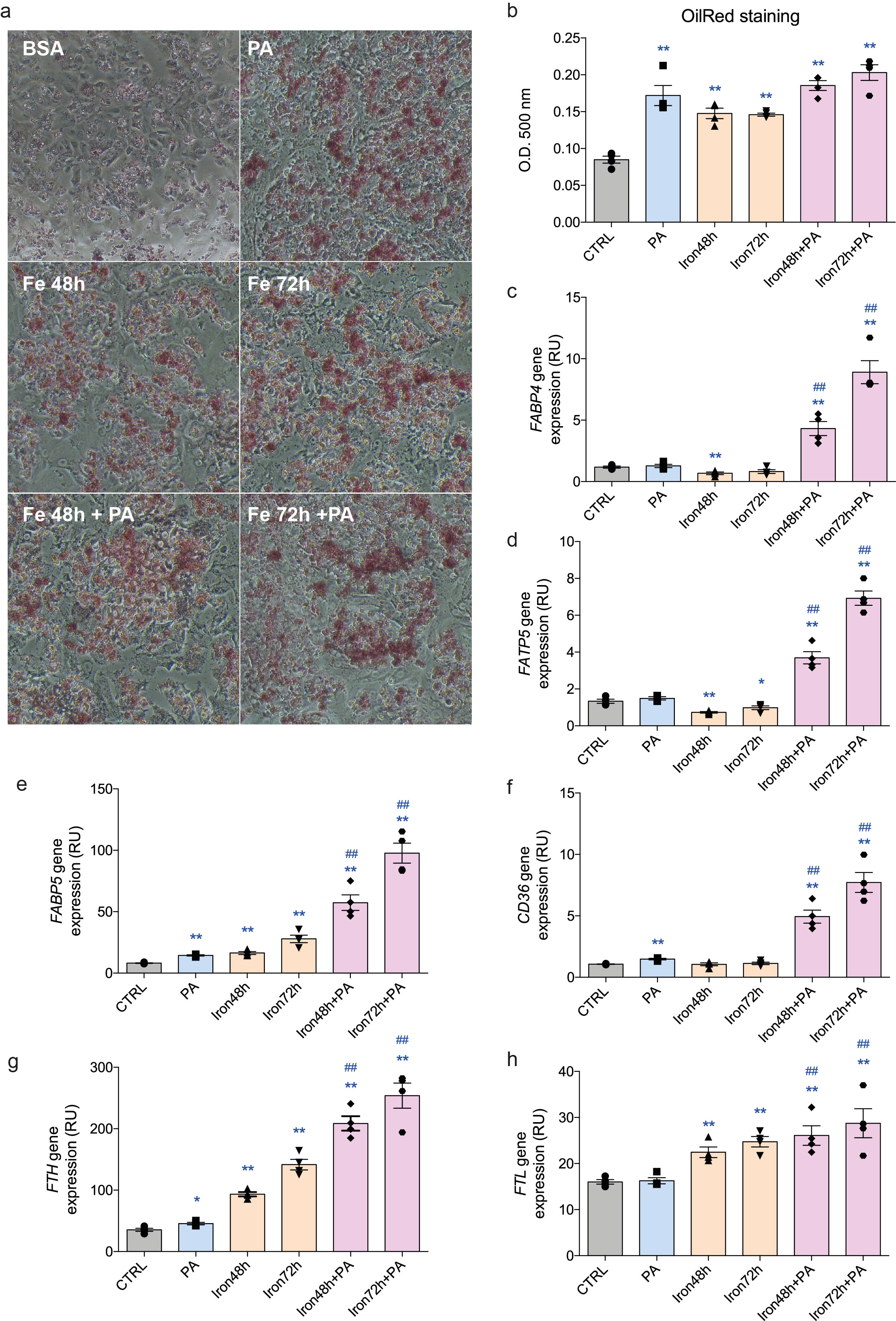

Supplement: Supplementary file 13 — Additional file 12: Figure S7. Iron supplementation leads to triglyceride accumulation and induces the expression of lipid and iron metabolism genes in primary human hepatocytes. a) Micrographs of primary human hepatocytes stained with Oil Red-O (representative images are from n = 4 independent batches). b) Quantification of lipid accumulation. O.D., Optical Density. c-h) FABP4, FABP5, FATP5, CD36, FTH, and FTL expression in hepatocytes. Data are mean ± SEM. Comparisons by one-way ANOVA. *p<0.05, **p<0.01, ***p<0.001 compared to control group based on t-test. #p<0.05, ##p<0.01, ###p<0.001 compared to PA group based on t-test. Ctrl, control group; PA, palmitic acid; Fe48h, pre-treatment iron 50μM for 48h; Fe72h, pre-treatment iron 50μM for 72h; Fe48h + PA, pre-treatment iron 50μM for 48h + palmitic acid 200μM for 24h; Fe72h + PA, pre-treatment iron 50μM for 72h + palmitic acid 200μM for 24h. [file 40168_2021_1052_MOESM13_ESM.jpg]

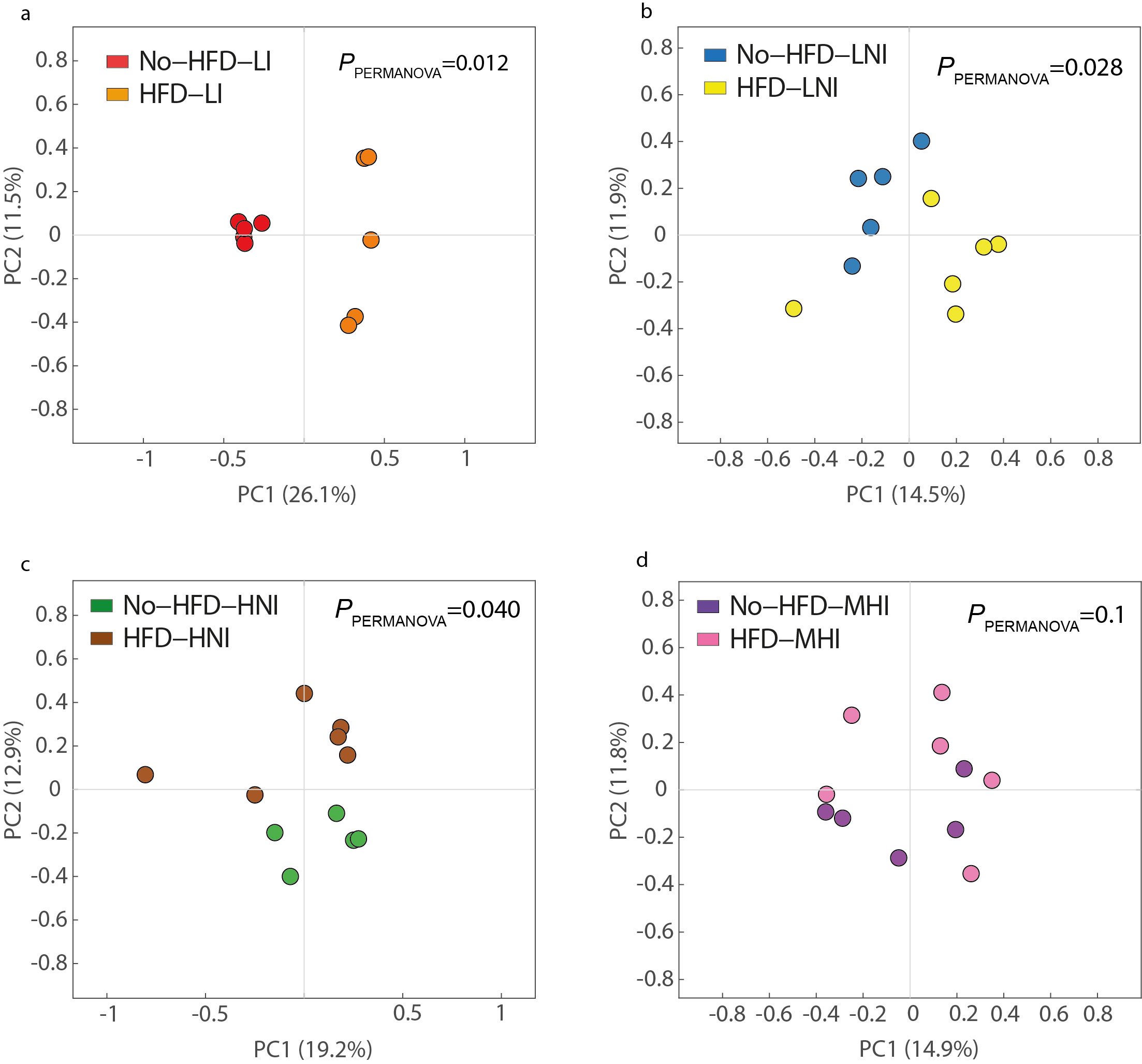

Supplement: Supplementary file 14 — Additional file 13: Figure S8. PcoA based on Canberra beta diversity comparing high fat diet (HFD) and non-high fat diet (No-HFD) for different iron doses. a) low-iron (LI) fed mice, b) low-normal-iron (LNI) fed mice, c) the high-normal iron (HNI) fed mice, d) moderately-high (MHI) iron fed mice. Differences in microbial composition were assessed by PERMANOVA analyses using the Adonis function in vegan R package with 999 permutations. [file 40168_2021_1052_MOESM14_ESM.jpg]
